# Seeing the unseen: Comparison study of representation approaches for biochemical processes in education

**DOI:** 10.1371/journal.pone.0293592

**Published:** 2023-11-06

**Authors:** Hana Pokojná, Barbora Kozlíková, Drew Berry, Simone Kriglstein, Katarína Furmanová

**Affiliations:** 1 Department of Visual Computing, Masaryk University, Brno, Czech Republic; 2 Walter and Eliza Hall Institute of Medical Research, Melbourne, Victoria, Australia; 3 AIT Austrian Institute of Technology GmbH, Vienna, Austria; Tallinn University: Tallinna Ulikool, ESTONIA

## Abstract

The representations of biochemical processes must balance visual portrayals with descriptive content to be an effective learning tool. To determine what type of representation is the most suitable for education, we designed five different representations of adenosine triphosphate (ATP) synthesis and examined how they are perceived. Our representations consisted of an overview of the process in a detailed and abstract illustrative format, continuous video formats with and without narration, and a combined illustrative overview with dynamic components. The five representations were evaluated by non-experts who were randomly assigned one of them and experts who viewed and compared all five representations. Subsequently, we conducted a focus group on the outcomes of these evaluations, which gave insight into possible explanations of our results, where the non-experts preferred the detailed static representation and found the narrated video least helpful, in contradiction to the experts who favored the narrated video the most.

## 1 Introduction

Visual communication of abstract scientific concepts is widely used in education [1]. The upheaval of technological advancement provides different types of visualizations that go beyond typical textbook diagrams, offering many ways of creating visual learning platforms [2]. However, illustrations and animations still remain the most available means of scientific communication. The compelling visual translation of processes and structures in science continues to be facilitated through medical art [3]. The need to use a suitable representation of different occurrences is essential as it yields higher success in scientific practice [4]. In biochemistry, the necessity of visually communicating biochemical processes is even more prominent, as these are not visible and must be represented accurately due to their abstraction. Previous research has shown the benefits of static images and animated videos in explaining concepts in biochemical and biomolecular fields, for example, in work by Goodsell et al. [5]. While medical and molecular art provides some artistic freedom, effective representations utilize a set of rules when using multiple media outlined in the Theory of Multimedia Learning [6] to create effective content for learning based on not overstimulating the sensory load. However, the requirements and preferences for representations differ between experts in the field and non-experts trying to grasp the context [7]. Therefore, communicators need to know their audience [8].

In our study, we explore the preferences of different audience groups for the different representations—static overview illustrations, animations, and their hybrid combination—by analyzing their benefits and shortcomings. We aimed to find out what kind of representation is most helpful in understanding the concepts in the complex process happening at the molecular scale. We chose the well-known adenosine triphosphate (ATP) synthesis [9] as a representative process in our study. The concept of ATP synthesis is commonly taught in biology courses at the high school level in Europe [10]. At the same time, it is a non-trivial and abstract concept that requires substantial effort to understand it. It consists of several sub-processes and involves multiple mutually interacting actors, and their precise appearance and mechanics cannot be easily captured and observed. Some of the difficulties of understanding the energy-making process also come from a lack of understanding of the molecular structure [11]. Other difficulties in understanding this process stem from not being able to see the connection between core chemistry and biological concepts due to lack of opportunity [12, 13]. These properties are typical for many abstract concepts and processes that commonly need to be communicated in education and research of natural sciences, such as biology, physics, or chemistry. As such, the outcome of this study, which focuses on the accurate depiction and effective retention of the process, can be generalized to other STEM sciences [14].

**Fig 1:**
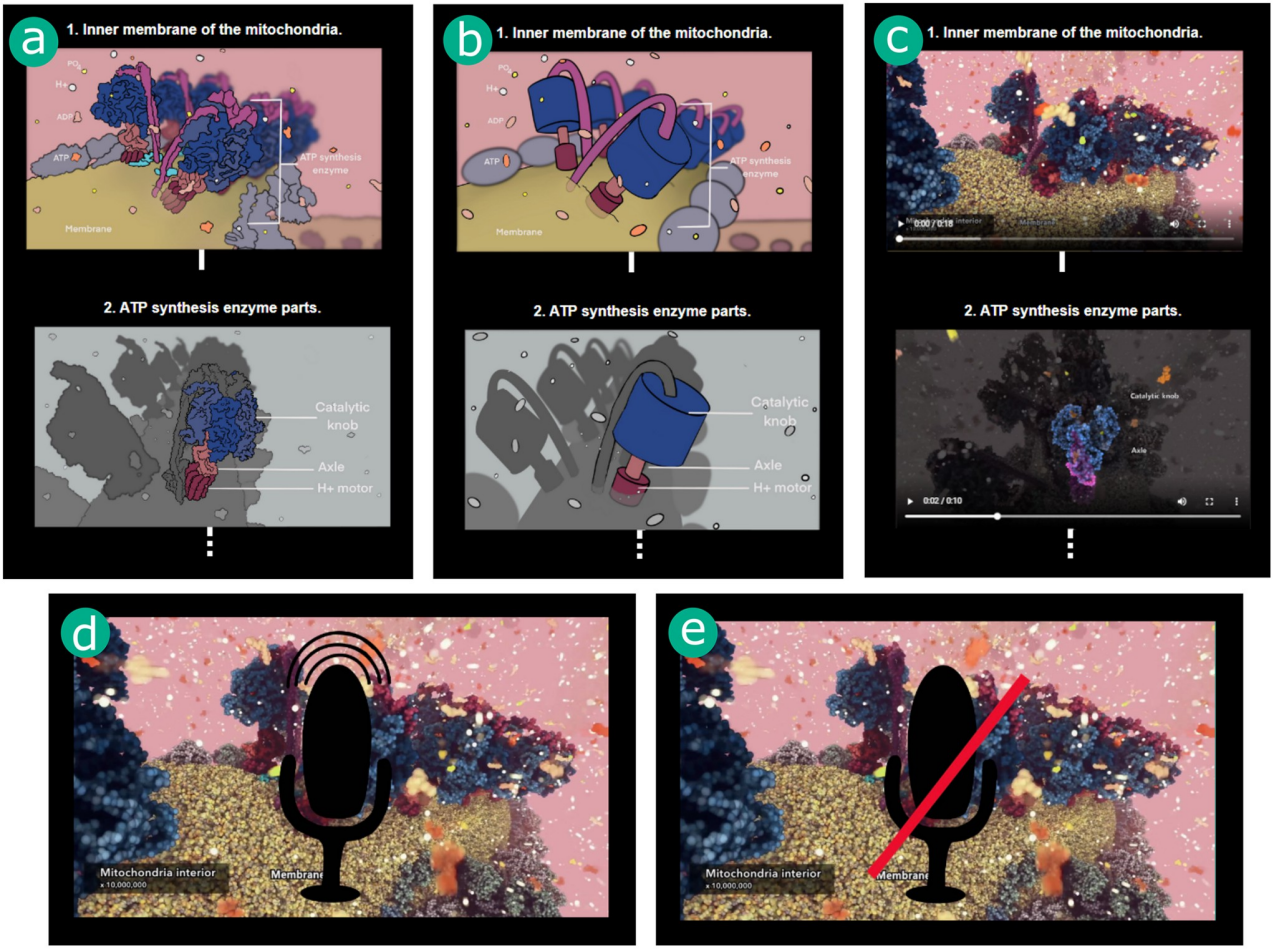
Types of representations. (a) Detailed Static, (b) Abstract Static, (c) Hybrid, (d) Narrated Video, (d) Video without Commentary.

**Fig 2:**
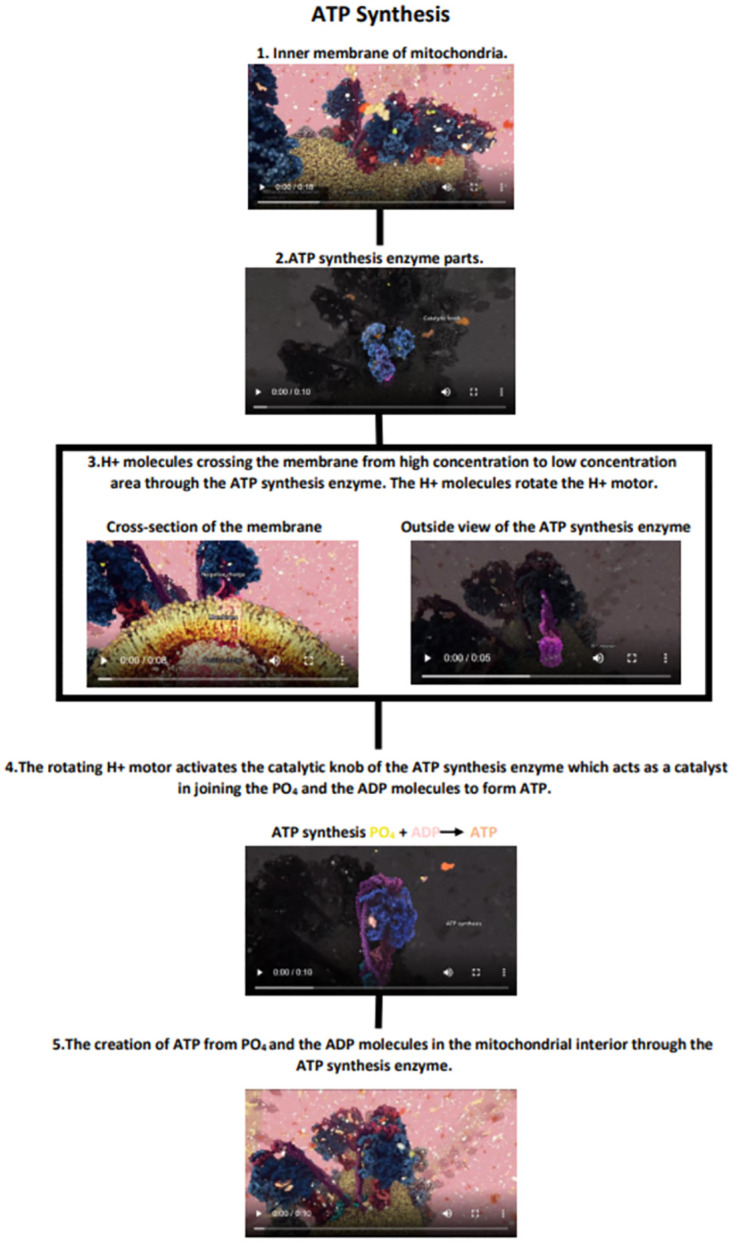
The arrangement of the overview representations. Hybrid representation showing the arrangement of the overview of the process and dynamic elements in the form of 4–11 sec. videos accompanied by written descriptions.

We designed five different representations of the ATP synthesis process (see Fig 1); (a) Detailed Static, (b) Abstract Static, (c) Hybrid, (d) Narrated Video, and (e) Video without Commentary, further referred to as Video. The Detailed Static, Abstract Static, and Hybrid representations depicted the process as a series of key steps of the process with textual descriptions presented in the flow-chart-like storyboard (see Fig 2), providing *overview* of the entire process. They differed in the way individual steps were represented—detailed static illustrations, simplified (abstract) static illustrations, and animated sequences, respectively. On the other hand, the Narrated Video and Video without Commentary presented the process in a single *continuous* video sequence, differing only in the presence of the auditory content.

The use and advantages of both illustrations and animations for the education and communication of science have already been studied previously. However, to our knowledge, no previous research combines an overview scheme of the entire process with animated media, such as our Hybrid representation. We hypothesized that this combination of the schematic representations outlining key parts of the process, with animations that communicate motion and mechanics of the process, would be most useful from all the representations. To evaluate the benefits and drawbacks of the Hybrid representation against more standard formats, such as static illustrations and videos, we conducted a series of studies. Our studies also aimed to provide more insight into which commonly used visual aids are useful and effective in learning about abstract biochemical processes and what preferences different audience groups have. Our main focus was to find the best way to represent these processes to high school students, where the findings could be further applied to related areas of education. We also focused on finding the best representations for expert use, whether it be for the dissemination of work in the research or education world. We aimed to answer the following questions:

- Is it more beneficial for learning to view the biochemical process in a continuous or an overview scheme showing the main phases of the process?
- Is there a difference between user groups with varying levels of knowledge in preference for static or animated representations?
- Is there any preference for abstract or detailed representations between the expert and non-expert target audience groups?

Our findings can help teachers and creators of educational materials for different audiences choose an appropriate format of visual representations, considering aspects such as layout, content fragmentation, visual complexity, and aesthetics. We believe that our gained insights can contribute to a better design of learning materials for biochemical processes beyond the ATP synthesis and extend the process presentation to other domains, for example, biophysics or medicine.

Our paper is structured in the following way. After summarizing the existing works related to our study, we introduce our methodology for designing the different representations and the study itself. Then, we summarize and discuss the results and suggest potential future research directions.

## 2 Related work

To successfully carry out a study that aims to explore the comparison of different techniques showing dynamic processes on a molecular level, we have looked at previous research and theories that address similar goals. The following section shows the research already undertaken into effective depictions of biomedical data and explains why we have chosen the representations we did for this study (Sections 2.1- 2.3). This includes research into illustrations and videos, abstractions of designs, and the benefits of the whole process overview. The second half of the related work states principles that should be applied in all educational representations and that we have considered in our study when creating and choosing our five representations (Sections 2.4–2.5). This includes the importance of storytelling in science and Multimedia Learning Theory [15]. Concepts from the research discussed in this chapter have inspired the representations we decided to use in this study and how we created them.

### 2.1 Illustrations and videos

The standard means of communicating science used to be through medical illustration. Medical illustration is a widely used technique, for example, in education and patient outreach, where specialist illustrators use various methods to effectively communicate medical occurrences [3]. These range from depicting events from overall anatomy to detailed interaction of biochemical reactions [3]. Similarly, small-scale molecular illustrations are very helpful in education, research, and dissemination [16]. According to Adnan et al. [17], subjects such as biochemistry benefit greatly from illustrations as they increase understanding and motivation and create a positive and enjoyable learning experience when used to teach the bio-molecular processes such as Krebs Cycle and electro-transport chain. This is also shown by the widely used software BioRender.com (2022), designed specifically for biochemistry experts to visually help explain processes and structures invisible to the naked eye and remove the limitation of needing graphic design experience [18]. Visualizations are also favored because they help understand unseen processes and relationships difficult to describe [16]. As Iwasa [19] has stated, visualization in biomedical fields requires results to be more understandable. Other than education and dissemination, the new findings may be based on understanding specific structures of certain molecules; hence their visual representation needs to be accurately shown. Iwasa [19] also described the move from hand-drawn images to professionally made renders and even the benefits of visual metaphors in education.

Molecular animations have an advantage over illustrations as they show continuous change. Yet, the need for dynamic depiction depends on the audience and the objective to be communicated [20]. The same research also suggested that more complex animations led to higher scores on tests, which meant that different levels of complexity and abstraction might be useful in different types of learning. It was also demonstrated that three-dimensional (3D) animations help explain complex bio-molecular assemblies [21]. 3D animations have advantages as they can use accurate 3D models of proteins and other molecules provided by collections such as the Protein Data Bank [22], or collected structural data obtained by researchers. Moreover, the animations effectively show dynamic, temporal, and spatial data [23]. Biochemical animations can be created with different levels of accuracy and abstraction using 3D modeling software with specially designed add-ons, for example, MolecularMaya (2020) or open-source Bioblender (2022). There is also a possibility of showing static data with animation using different camera angles to help spatial understanding [24]. Mansor et al. [25] have reviewed that animations are effective learning tools because of the audio, visuals, and design. They also concluded that animations are useful in helping the viewers visualize things that are not visible and are difficult to visualize without aid. Other media include molecular simulations in augmented and virtual realities [26]. In addition to different means of displaying visualizations, different types of visualizing the unseen molecules based on their structures and their use for further research in biochemistry are discussed by Kozlíková et al. [27]. As summarised here, technology provides many ways of creating educational material. However, creating the material is only the first step in making an effective educational tool, which is the scope of our research. Another aspect is to consider the right way of approaching the storytelling behind the visual content.

### 2.2 Abstraction in scientific representations

Once the media is chosen and the story decided, one must also consider stylistic choices and aesthetics of the output. Abstracted images have been proven to have the benefit of lending more insight into data [28]. This is further supported by Andrews [29], who refers to abstraction as a tool to remove ‘visual garbage’ in medical illustration. As Falk et al. [30] suggest, a busy environment of the cell can include cues that help reduce visual complexity, such as signal molecules. In practice, the preferences for the level of abstraction may differ between expert and non-expert target audiences. The exploratory study by Garrison et al. [31] employed expert and non-expert participants to evaluate different types of data visualization across scales of well-known biomedical processes seen through the micro, meso, and macro lenses. The results have shown that experts and non-experts overlap in abstraction preferences, mainly that processes should not be too realistic or abstract. There are several explanations for this; experts do not need detailed representations because they already possess the necessary knowledge to understand the concept, while non-experts prefer more straightforward representations to limit visual information overload. In contrast, previous research specifically aimed at students learning from four levels of detail in molecular animation showed that the more complex molecular videos were, the better students performed on tests [32].

### 2.3 Whole process overview: Graphic medicine and data comics

One of the questions we aim to answer with this research is whether a continuous presentation of data, as in the video, is more effective than an overview of the entire process, which allows the viewer to skim-read and then return to the place of interest. One example of using an overview of information is data comic visualization, which was proven to be effective [33]. Comics are defined as ‘sequential art’ and are relevant to the text within the comics [34]. Alemany-Pages et al. [35] discussed the use of science comics and the technique of using relatable metaphors for abstract processes or their components to help students learn and understand science concepts. Another successful example of a pictorial overview of scientific concepts is the ‘Stuff of Life: A Graphic Guide to Genetics and DNA’ by Schults [36]. Besides using comics for data presentation among scientists [33], they can also be utilized for communication with the public and patients [37]. Comics are a convenient medium as they provide visual and textual content, along with sequential information, which allows for quick recall navigation [38]. This medium is also helpful for different levels of reading, whether skimming or close reading because it provides access to details through an overview. These visual overviews are also often used for storyboard creations, serving as templates for detailed animations [39].

### 2.4 Scientific storytelling

Storytelling is essential to learning about abstract processes, such as biochemical processes inside cells. Botsis et al. [40] suggested that visual scientific storytelling increases knowledge. A well-thought-out and presented visualizations often use the combination of education and art [41]. Bridging these two areas effectively requires the viewers to train specific cognitive skills [41], and if done successfully, biochemical visualizations are helpful learning tools. Ma et al. [42] have suggested that good representations tell a story with enough context to visualize the concepts, go at a pace that the viewers understand, and explain how the main actors interact while keeping the viewers’ attention. Some of the principles of scientific storytelling include the introduction of ‘actors’ and a summary of events at the conclusion of the scientific process [43]. Making storytelling the main focus of the learning process has been shown to be effective in education and public outreach [5]. An example is the successful annual PDB-101 Video Challenge, for high school students, with varying topics presented each year. The student participants are asked to depict the molecular process in a video story format.

### 2.5 Multimedia learning theory

To create an effective educational tool, one must follow rules that balance enough provided information to explain the process and not overwhelm the viewer, which hinders the learning process. Mayer and Moreno [15] proposed the Theory of Multimedia Learning based on dual-channel processing. The separate networks work together to understand pictorial and verbal stimuli, and each has a limited capacity. When a viewer is overstimulated, they can ‘fill up’ each of these channels to the point where they cannot learn. Learning is, in this case, presenting information that can be recalled at a later time from Long-Term Memory. Mayer and Moreno [15] also present ways to reduce cognitive load by providing a formulation of using words and visual and auditory stimuli to make the learning most effective. They identified issues and their possible solutions to them. Five types of overload were identified, and nine ways of reducing them were listed. A set of principles was also proposed [6], specifically focusing on making animation an effective learning tool. A practical application of these principles in molecular animation was described and based on an observational study of genomic and molecular animations by Patterson et al. [44].

## 3 Representations design

ATP synthesis is the process through which living cells convert adenosine diphosphate (ADP) molecules into ATP energy inside the cellular organelle mitochondria, often referred to as the ‘powerhouse of the cell’ for this reason. It is one of the most critical yet complex processes in living cells. The ATP synthesis was purposely chosen as the bio-molecular process to be represented in this study because it is commonly internationally taught at the high school level as part of the biology curriculum [10].

Illustrations and videos are widely used to help explain the complexities in the biomedical field, as mentioned by the recent research above. The efficiency in showing movement, spatial relationships, and model accuracy is the reason we chose continuous representations. We have also decided to test the Video and Narrated Video between each other because of the benefits stated by Mansor et al. [25] and to test for audio impact. Our static representations, the Static and Abstract Illustrations, were chosen because of the benefits of illustrative techniques. These techniques have been used in science education for a long time and helped people visualize abstract concepts. The illustrations and videos each have their benefits, and hence we decided to create a new representation, the Hybrid, which combines the static elements of layout with the written description as well as the movement of the videos.

**Fig 3:**
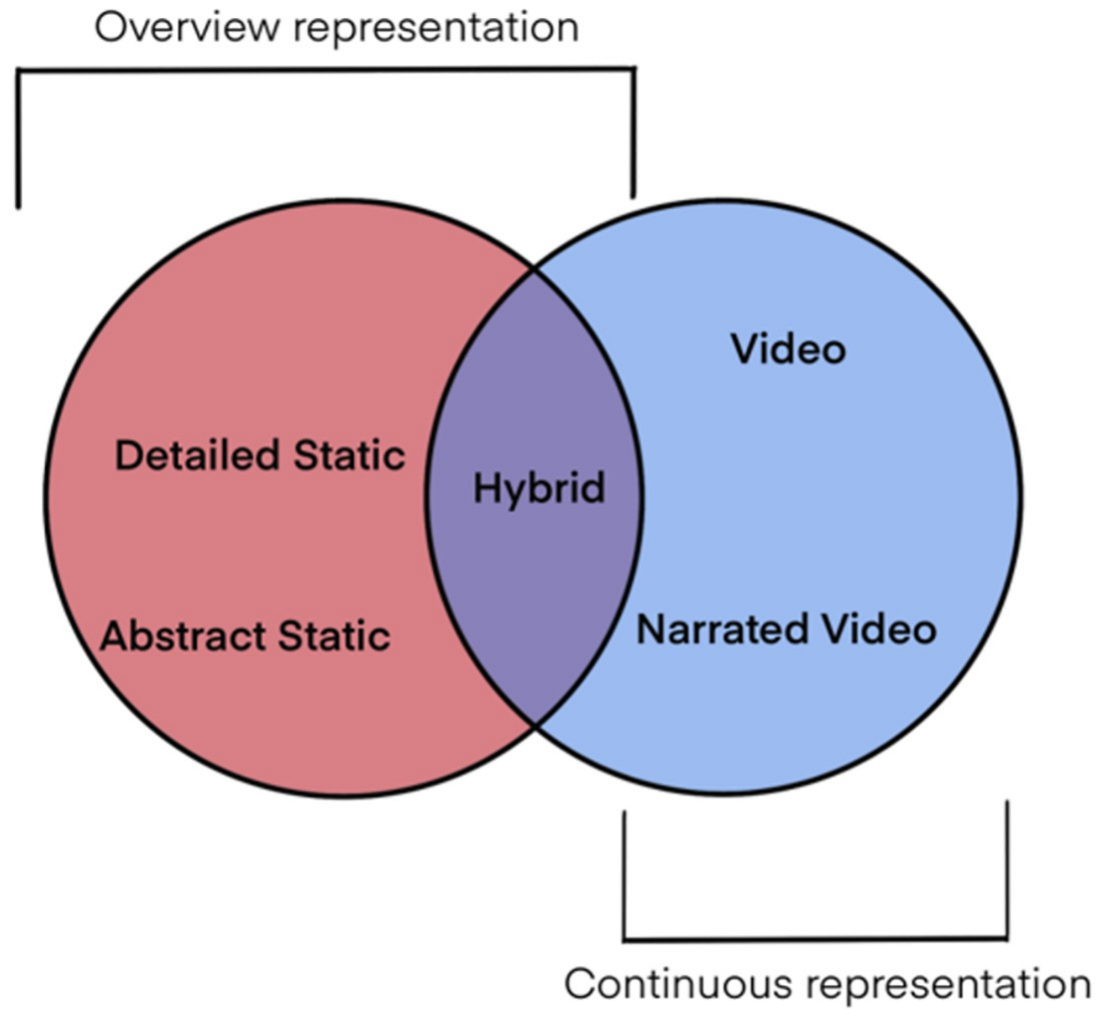
Division of the five visual representations. Division of the representations showing the overview (Detailed and Abstract Static) and continuous (Video and Narrated Video) representations and their intersection that is used in the Hybrid representation.

In our study, we use five different representations of the ATP process [9]; two static overviews utilizing flow-chart-like storyboard layout, two continuous videos, and one hybrid representation—the visual representation of this categorization can be seen in Fig 3. The static representations included Detailed Static (Fig 1A) representation and Abstract Static (Fig 1B) representation, designed to evaluate the aspect of abstraction in the representations. The continuous representations included 3D dynamic animation with background music known as the Video (Fig 1E) and 3D dynamic animation with music and narration presented as the Narrated Video (Fig 1D), included to evaluate the need for narration with dynamic animations. Finally, the Hybrid (Fig 1C) representation was designed to combine the continuous elements with an overview of the whole process. All of the representations can be seen in S1 File.

These five representations targeted our research questions. The benefits of each representation and why these specific representations were chosen are covered in the previous section containing related work. Previous studies have also shown that combining different learning aspects into one does not necessarily lead to higher retention [15], thus we have evaluated them in separate representations. This meant we created versions to compare representations that were already proven beneficial (videos and illustrations) against each other, as well as created a new representation that combined the benefits of the overview approach with animations.

One aim was to test abstraction level understanding with the knowledge level of the participants. As discussed, research states that abstraction may be beneficial in some cases [28]. This was the driving force behind choosing Detailed Static and Abstract Static representations to compare abstraction each other. This allowed us to observe which level of abstraction is preferred by people with higher versus lesser biochemical knowledge in the context of ATP synthesis.

The Multimedia Leaning Theory [15] guided us in creating the representations so that they are divided into understandable and easily retainable parts in the overview approach.

Because of the importance of effectively telling a story about the process, we considered storytelling elements in the representations we chose and created. This included the layout of the story, descriptive text of the story, as well as visuals, and division into appropriate parts in the storyboard overview approach.

The five versions of the ATP process were based on a 3D animated educational video by a professional biomedical animator [45]. All of the versions needed to contain critical points in the ATP synthesis process, as well as show the movement of the main actors, such as the flow of *H*^+^ protons and *H*^+^ motor rotation. In addition to the main actors, based on the principles of effective storytelling [43], our representations also included an opening with an introduction of the setting and main actors as well as a conclusion summarizing the process.

The original video with the narration was cut to be a minute and a half long and to encompass just the process of ATP synthesis from the beginning to the end and was used as the Narrated Video representation. The animator also provided the version that was used as the Video representation, which contained sound effects and visuals identical to the Narrated Video but did not contain the voiceover.

The parts of the video representing the critical moments in the ‘story’ of ATP synthesis were used as the basis for the remaining representations employing storyboard overview layout (Detailed Static, Abstract Static, and Hybrid). These representations were arranged in the same manner and showed the same order of events with the same textual description. For the static representations key video frames were carefully chosen to be able to convey dynamic movement even in static conditions. They were further enriched by labels, arrows, and descriptions in text using action verbs that describe the movement of the actors in the process. As such, each of the selected frames could also serve as a standalone representation. For the Detailed Static representation, the selected keyframes were recreated as illustrated images that resembled the models from the original animation in complexity (for example, folds on the enzyme). The Abstract Static version was based on detailed illustrations simplified to basic three-dimensional shapes, such as cylinders, spheres, and ellipses. Both of the static representations used the color scheme from the original video.

The Hybrid representation was made by creating short animations (4–11 seconds) from the original video provided by the animator showing the dynamics of the critical moments of the ATP synthesis process. This representation was designed to combine the best components from the overview storyboard approach—outlining the entire process—with dynamic components of the video representations—communicating the motion.

At the same time, the Hybrid representation used interaction by letting the learners start the short animation at each key step in order to view the next part of the ‘story’. They could also replay the animations as it suited them. This differed from passive observation of a static image or a video. While the video representations could also be paused and re-winded, we expected that these interactions would be less frequent in the fully continuous representations.

The representations were made using the video editing program Adobe After Effects (2020) and Procreate (2020) drawing application.

## 4 Methodology

Our comparison study aimed to collect data that indicate preferences for different types of representations and their properties, such as level of abstraction and continuity. These data are intended to help choose a proper way of abstract data representations in biomedical sciences for educational purposes. We expected that people with different levels of expertise would prefer different types of information visualization as some contain more information than others [7].

Our study collected quantitative and qualitative data through two iterations and a focus group. In the first part, we conducted two independent online surveys with different target groups, one with non-expert participants (n = 60) and one with experts with backgrounds in biology and data visualization (n = 9). These surveys aimed to assess and compare the educational potential of the representations and collect feedback and preferences from different target groups. Subsequently, we conducted a focus group on the survey results with experts (n = 5) with specific expertise in biology, visualization, design, and education. The qualitative data from open questions from both iterations were later analyzed for themes and ideas with Qualitative Content Analysis [46].

The research presented in this paper was conducted at Masaryk University, Czech Republic in accordance with the Czech law. We have conducted two anonymous online surveys with participants over 18 years of age. No sensitive personal data were collected in these surveys. To further evaluate our results we have also conducted a focus group. The participants of the focus group were over 18 years of age and provided written consent by signing an informed consent form regarding the procedure and further processing of the collected data. The results presented within this paper were fully anonymized and do not contain any personal or sensitive information about the participants. According to Czech law, ethical approval is required only for research in the field of biomedicine/research involving biomedical techniques (e.g., MRI, the sampling of blood or other biological materials, measuring biological parameters), or research involving underage participants. With regards to this law, we were not required to obtain an ethical approval.

### 4.1 Participants

The different surveys and a focus group allowed us to collect qualitative and quantitative data from users with different knowledge levels and experience with biochemical visualizations.

- The experts were needed to suggest improvements and point to valuable components of the representations since they have experience working with abstract processes.
- The non-experts’ input allowed us to see how good already existing representations are and find out first-hand what other components would facilitate more accessible learning.
- The experts from various backgrounds provided insight into the explanation of previous results and future research from different perspectives and also added validation to previous findings.

#### 4.1.1 Non-experts

The non-expert participants were reached through convenience sampling (university mailing lists and social media). They included predominantly university students from study programs related to the focus of the study, such as biochemistry-related fields and visualizations, and students of unrelated fields, such as law, aircraft design, and urban studies. The variety of students with related and unrelated backgrounds was done purposely to avoid bias in learning about the biochemical process and to gain feedback from students learning biological subjects that would benefit from these representations. The distribution of participants’ capabilities and interest in biochemistry is similar to the distribution of these factors in students in class. They consisted of an international sample from 12 countries; Austria, Czechia, France, Lebanon, Netherlands, Norway, Palestine, Portugal, Serbia, Slovakia, United Kingdom, and the USA. Of these participants, 23 were female, 33 were male, 3 were non-binary, and 1 participant preferred not to say. Their age range was 20 to 43, with an average of 23.84. Two participants with visual impairment (mild deutian vision and deuteranomaly) were included in the result analysis as their responses were not statistically different from the rest of the non-experts. Participants were randomly given one of the five representations—each representation was assigned to at least 10 participants; Detailed Static n = 14, Abstract Static n = 12, Hybrid n = 10, Narrated Video n = 13, Video n = 11. A summary of the non-experts’ information can be found in S3 File.

#### 4.1.2 Experts

We invited two groups of experts to participate based on their backgrounds: visualization and biochemistry. These areas were chosen as the experts from these fields could give valuable insight into biochemical representations, either as the users or as the creators of visualizations of abstract scientific data. The experts were reached through our professional networks and included participants from six countries; Argentina, Austria, Czechia, Taiwan, Slovakia, and the USA. All participants in this group had normal vision. The summary of the expert description can be found in S4 File.

#### 4.1.3 Focus group

For the focus group, we invited participants with diverse backgrounds to help evaluate previous results and give insight into future research through semi-structured discussion. Participants included a high school biology and chemistry teacher (four years of experience), biological visualization researcher (ten years of experience), a visualization and human perception researcher (fifteen years of experience), a graphic designer (fifteen years of experience), and biochemistry researcher (twenty years of experience). All participants had teaching experience. The demographics of focus group participants are included in S5 File.

### 4.2 Study design

The first iteration of the study consisted of two online surveys, which can be found in S2 File. The online questionnaires were chosen to facilitate easier and faster outreach to a larger population of non-experts and experts. Both surveys were composed of two parts—participant information and representations questionnaire. In the first part of both surveys, we collected demographic information, such as nationality and age, as well as information about the participant’s level of expertise in biology and visualization and visual impairments, tested by an online color blindness test. The surveys were anonymous, and no sensitive information was collected.

We conducted a pilot test for each survey with two participants to estimate the timing and uncover potential issues. Subsequently, the surveys for experts and non-experts ran independently and lasted approximately three months each.

The results from both groups were analyzed and presented in the focus group as points for discussion alongside the participants’ own opinions on each of the representation, their use, critique, and future improvements.

#### 4.2.1 Non-expert survey

Each non-expert survey participant received a questionnaire with one randomly selected representation. The questions in the survey were the same for all non-expert participants. We included two categories of representation-related questions—content questions designed to objectively test the participant’s understating of the depicted biochemical process, and questions regarding subjective preferences and impressions about the given representation. The content questions were open-ended, with short unambiguous answers, such as ‘Which two molecules react to make the ATP molecule?’ To evaluate the understanding of the order of events of the biomedical process, participants were given the task of ordering the main events using the drag-and-drop tool. A five-point Likert scale was used to measure the subjectively perceived usefulness of representation in terms of communicating movement and sequence of events. Furthermore, participants were asked to describe the representation by selecting options from a list of positive (informative, accurate, pretty…) and negative (excessive, confusing, distracting…) attributes which provided us with the participants’ impression of the visuals [31]. Finally, the survey also included open questions regarding likes, dislikes, and suggested improvements.

All five representations were designed so that the questions about the ATP synthesis in the non-expert group could have been answered from any of the representations. Participants were allowed to return to the representations as often as they liked and were asked in the end to report how many times they looked back. The whole survey was designed to take up to 30 minutes.

#### 4.2.2 Expert survey

The expert questionnaire included all five representations and specific questions regarding their usefulness and educational value. Participants were again allowed to return to the representations as often as they wished. In the survey, the experts were asked to select the most and least helpful representations in general and representations that best communicated movement and sequence of events. For each of these questions, they were also asked to provide reasons for their choices, for example: ‘Which representation was most helpful in understanding the movement of molecules and enzyme and why?’ The questionnaire also included closed questions asking participants to choose from a list of positive and negative attributes to describe each of the representations (same as in the non-expert survey) to help us understand their perception of them [31], and the option to provide additional feedback and suggestions for improvements. Finally, a series of open questions regarding the level of abstraction, continuity, and sequential overview were included. Parts of our questionnaires were based on a preference study used by Bear et al. in their perceptual evaluation of visualization techniques for cerebral aneurysm anatomy [47]. The survey was designed to take up to 60 minutes.

#### 4.2.3 Focus group session

After the responses were collected from both online survey groups, the data were analyzed and compared. A focus group method was chosen as it has the advantage of showing immediate results and time effectiveness and was based on Courage & Baxter [48] technique. A week before the focus group, participants were sent online materials with all five representations to get familiar with them. They were informed that these representations and their utility for education would be discussed within the session.

The focus group took place in a seminar room with a table, chairs, whiteboard, and a TV monitor where the representations were displayed again. Alongside the participants, there were present one moderator, one note-taker, and a camera recording the discussions with the participants’ consent. The session lasted about 120 minutes and consisted of two parts. In the first part, a semi-structured discussion about individual representations was held, with participants offering their opinions, suggested improvements, critiques, real-life use, and possible future research directions. In the second part, participants were presented with the outcomes of the previous surveys and discussed the reasoning for the results and their implications.

## 5 Results

The combination of qualitative and quantitative results was collected from surveys with students, visualization and biochemistry experts, and a focus group.

### 5.1 Data analysis procedure

The results from the non-expert survey were split into three parts: subjective scores, objective scores, and qualitative feedback. We have used one-way ANOVA and between-subject t-tests to calculate statistical significance between the test groups. The subjective scores were computed as averages from the users’ subjective ratings indicated on the five-point Likert scale. To obtain the objective scores, the non-expert results were analyzed by correcting the right and wrong answers in the content and order-of-events questions, which yielded correctness percentages for each person and each group. We then calculated the overall objective score by combining the score averages of the content questions regarding the movement and chemical actors with the average score for the order questions. Two participants (one from the Hybrid representation group and one from the Abstract Static representation group) were eliminated from the quantitative analysis because they indicated that their drag-and-drop tool in the order questions was not working, which was also seen in the raw data results because only these two participants had the order of events in the default position. Including these two participants’ content scores without the order score to make an overall average would significantly impact the overall results, hence, their quantitative elimination. However, the qualitative feedback for open questions from these two participants was still used, as their responses on what they liked and disliked about the representation contributed to the evaluation. In the evaluation of open questions, we looked for repeated themes, such as what the participants liked and disliked about each representation in the open comments. The responses for keywords were counted for each representation to get an idea of the general impression of the participants.

The expert group’s answers were qualitative and divided into themes about the preference of representation (most and least liked), critique for each respective representation, their comparison, and the potential use of each representation in practice. The discussion from the focus group was recorded, and upon re-examination, themes were established. This involved positives and negatives about each representation, each of the representation’s potential improvement and critique, suggestions for its use, and additional comments.

Qualitative Content Analysis [46] was used for analyzing qualitative answers in the two online surveys and the focus group discussion. The procedure consisted of finding themes in the data. The higher frequency of a certain theme amongst the participants indicated more importance. This was particularly useful in finding the main benefits and shortcomings of the visualizations. These themes are listed in the Results section.

For the final results, the objective content scores of non-experts were used to determine the order of representations’ helpfulness. This order was compared to the order of helpfulness of experts’ results determined by their answers of which representation is their most and least favorite one.

### 5.2 Non-experts

The results were measured by quantitative overall knowledge scores by the participants and their ratings of different attributes of the representations (such as rating perceived helpfulness and order versus content questions. The objective scores were the Overall score calculated from the content and order questions.

#### 5.2.1 Overall score, content, and order questions

The highest overall objective score (71.73%, SD = 24.96) and some of the qualitative comments in the open-ended questions indicated a preference for Detailed Static representation. The Narrated Video was the least helpful representation based on the objective score (46.64%, SD = 20.29). The Hybrid representation was the second most helpful, with 69.90% and SD = 21.22, followed by Abstract Static (64.39%, SD = 24.32). The second least helpful representation was Video representation (53.41%, SD = 23.63). The One-way ANOVA test has shown a significant difference (p = 0.0396, F = 2.714) between the groups. The quantitative results clearly favor the storyboard overview approach, as the representations with this design were the three with the best results. The t-tests show that the Detailed Static representation was statistically different from the Narrated Video (t = 2.987, critical value = 1.708, df = 25) and Video (t = 1.959, critical value = 1.717, df = 22). There was also a significant difference between the Hybrid and Narrated Video (t = 2.71, critical value = 1.74, df = 17) and no significant difference with Video (t = 1.733, critical value = 1.734, df = 18).

The content questions that measured the understanding of the process asked about specific chemical actors and understanding of the movement of molecules and enzyme parts. The one-way ANOVA test showed no statistical difference in the answers to content questions between the groups (p = 0.059, F = 0.71). The Detailed Static group achieved the highest score on content questions (64.88%) and the lowest score for the Narrated Video (52.56%).

According to the one-way ANOVA, the order-related questions showed a significant difference between the groups (p = 0.048, F = 2.57). The highest scores for order of events were achieved by participants in the Hybrid group (86.11%) and the lowest by the participants with Narrated Video (46.15%).

The summary can be seen in Table 1, where the statistical significance in the one-way ANOVA tests is indicated by an asterisk, while statistical significance in the t-tests is indicated by superscript letters [49].

**Table 1:** Summary of results by non-experts showing the order and movement average rating score (subjective measure), content and order scores achieved by participants (objective measure), calculated overall score (objective measure), and themes of positive and negative comments left by the participants in open questions. One-way ANOVA was performed between the representations in the content score, order of questions, and overall score. The significance is indicated by ‘**’ signifying p ≤ 0.001, ‘*’ signifying p ≤ 0.05, and ‘ns’ indicating p > 0.5. T-tests were carried out in the overall score questions between the representations and the statistical difference is indicated by letters [49]. The green color highlights the highest score in each category, and the red color highlights the lowest-scoring representation in each category.

	Order (rating)	Movement (rating)	Content score—ns (in %)	Order score * (in %)	Overall score * (in %)	Positive comments	Negative comments
**Detailed Static**	3.79	3.42	64.88	78.57	71.73^*a*^	step-by-step presentation; arrows in images and labels; not visually cluttered	too detailed; too much information
**Abstract Static**	4.08	3.58	60.61	68.18	64.39^*abc*^	images helped to complete the task; simple style	confusing; too simplistic; hard to distinguish between molecules
**Hybrid**	3.50	3.30	53.70	86.11	69.90^*ab*^	simple steps; colors	distracting music; hectic, too many details; most information from text; distracting animations; no narration
**Narrated Video**	2.62	3.39	52.56	46.15	46.64^*c*^	realistic; cool; motion; music; written and narrated	too fast; dramatic; subtitles wanted; chaotic; busy; difficult to understand without biochemistry background
**Video**	2.36	2.91	59.09	50.00	53.41^*bc*^	realistic; sounds; colors	dramatic music; too many things happening; going back and forth in the video; difficult to understand without biochemistry background

#### 5.2.2 Perceived helpfulness

In the subjective measurement, non-experts were asked to use a five-point Likert scale (5 being the best and 1 being the worst) to rate how helpful the respective representations were in conveying movement and order of events. For movement, Abstract Static representation was considered the best (3.58), and the least helpful was Video representation (2.91). In the perception of conveying the order of events, Abstract Static (4.08) was perceived as the most helpful, and Video (2.36) was perceived as the least helpful. The summary can be found in Table 1.

#### 5.2.3 Keywords attributes

Participants were asked to tick keywords associated with their representation in a closed question showing options with checkboxes. Out of the list of a mix of positive and negative keyword attributes, the Detailed Static representation was considered the most detailed (n = 11) and informative (n = 10). Abstract Static representation was considered simplistic (n = 7) and informative (n = 6). Hybrid representation was associated with pretty (n = 6), detailed, and distracting (n = 5 in both). The Video was perceived as confusing (n = 9), distracting, and detailed (n = 5 for both). The Narrated video was seen as distracting (n = 10) and detailed (n = 9).

#### 5.2.4 Feedback questions

Based on the provided feedback, non-experts appreciated the step-by-step guide, the arrows in the images, and the fact that the Detailed Static representation was not visually cluttered. At the same time, the comments included ‘there was too much detail’ and ‘too much information’. The Narrated Video was described as too fast, dramatic, chaotic, and busy. The positive responses included appreciation for realism, motion, music, aesthetics, and the fact that it had written and narrated content. The summary of results can be seen in Table 1.

#### 5.2.5 Open feedback

Qualitative Content Analysis [46] was used to look for patterns and most used phrases and words describing the representations. The content was divided into positive and negative feedback. The themes were then further divided into more categories based on the frequency they were mentioned. These included colors and design of the representation, comments on music, division, continuity of the representation, and realism. In the open answers, the non-experts also commented, in all five representations, that they liked the color and did not appreciate the white text on the black background, causing head and eye aches. Participants noted that they did not appreciate the aesthetics of guiding lines in the Static and Hybrid representations. A summary of participants’ comments about the most and least favorite features, including additional comments, is available in Table 1.

#### 5.2.6 Re-visiting material

Participants were allowed to return to the representations as often as they liked. Overall, the people who returned 10+ times had the highest overall score (68%), followed by people returning 1–5 times (63.4%). Participants who returned 6–10 times had the second lowest overall score (60%), and the people who did not re-visit the representation at all had the lowest score (55%). Most participants went back 1–5 times (n = 27), followed by 6–10 times (n = 24), some did not go back at all (n = 4), and some went back 10+ times (n = 5).

*5.2.6.1 Detailed static*. People who did not go back at all (n = 2) had the lowest overall score at 30% of correctness. People who went back 10+ times (n = 2) had the highest score in correctness at 80% overall.

*5.2.6.2 Abstract static*. People who went back 6–10 times (n = 7) had the lowest overall score of 57.14%. People who went back 1–5 times (n = 5) had the highest score in overall correctness at 73.33%.

*5.2.6.3 Hybrid.* People who went back 6–10 times (n = 3) had the lowest overall score of 60%. The person who did not go back at all (n = 1) had the highest score of 80%.

*5.2.6.4 Narrated video.* Person who went back 10+ times (n = 1) had the lowest score of 20%. The person who did not go back at all (n = 1) had the highest score of 60%.

*5.2.6.5 Video.* People who went back 6–10 times (n = 6) had the lowest overall score 41.67%. People who went back 1–5 times (n = 5) had the highest score in overall correctness of 40%.

The results partially confirm our assumption that the non-experts will reach better scores when revisiting the representations. However, the correlation is not as strong as expected, indicating other factors are worth investigating in the future.

### 5.3 Experts

In the expert survey, participants selected their most preferred representations in general and for specific tasks such as communication of movement and order of events. Additionally, Qualitative Content Analysis was used to look for themes and ideas about each representation in the open feedback question. These themes helped us identify the experts’ reasons for preferring or disliking each of the representations and whether a *continuous* video or segmented *overview* approach to viewing biochemical processes is more beneficial. The results have unanimously shown that the experts’ preference lies in the moving animated representations (n = 5 for Narrated Video, n = 4 for Hybrid, and n = 3 for Video). The Narrated Video representation was also voted the best (n = 5) in explaining the biochemical process’s movement and order of events. In qualitative feedback, it was reported to be the most descriptive and clear when describing the process without switching between content, showing dynamic changes and dynamic movement. One participant mentioned that it was almost overloading. The least preferred representation among the experts was the Abstract Static representation (n = 1) which was described mainly as too simple to facilitate deep understanding. The experts expressed concern that it was simplified to the point where it could be misleading. On the other hand, the positive remarks regarding this representation included that it could be good for teaching basics. The summary of the expert feedback can be found in Table 2.

**Table 2:** Summary of results by experts showing how many participants chose the representation as best for overall preference, the given task (order of events and movement in the process), and the themes based on positive and negative comments for the representations. The preferences are shown in the number of experts’ votes to show the difference of favoritism between the representatives as shown by votes. The red color highlights the lowest-scoring representations, and the green color highlights the highest-scoring representations in each category.

	Overall preference (# of votes)	Order (# of votes)	Movement (# of votes)	Positive comments	Negative comments
**Detailed Static**	2	1	0		very confusing; visually crowded, without values, depth of field bad (bad 3D)
**Abstract Static**	1	2	0	good for teaching basics	too simple; not for deep understanding; oversimplified to the point of misleading
**Hybrid**	4	3	1	understanding movement of molecules, short enough video to concentrate	needs clearer transitions between clips
**Narrated Video**	5	3	8	realistic representation; space/movement; most descriptive; clearest; step-by-step without switching between content; dynamic changes; narration gives information	overload
**Video**	3	0	0		lot of change simultaneously; need to re-watch

#### 5.3.1 Keyword attributes

The keyword description helped us explain why participants in the different target groups find each representation helpful and unhelpful. The experts saw Detailed Static representation as too detailed and confusing (both n = 5), followed by distracting, informative, and visually unappealing (n = 4 for all). Abstract Static was perceived as simplistic (n = 7), followed by inaccurate, easy to read, and clear (n = 5). The hybrid was seen as pretty (n = 6), followed by excessive and informative (n = 4). The Narrated Video was marked as pretty (n = 8), followed by informative and detailed (n = 7). The Video was seen as pretty (n = 6) and detailed (n = 5).

#### 5.3.2 Learning and comparisons

Regarding the type of layout, a storyboard overview of the process, or a continuous video, more experts preferred the continuous video (5 out of 9). It was noted that videos are better for general understanding and showing dynamics. One participant out of the five mentioned that they would prefer a video they could stop for detailed learning and that it would be highlighted in the timeline. The other experts (n = 4) preferred an overview of the process for understanding. Of the four experts preferring the overview, two noted that they would prefer using their combination by first using the overview and then the continuous video representations.

For process understanding, experts deemed that Abstract Static representation (n = 6) is enough to understand the process compared to the Detailed Static (n = 2). One mentioned that this approach is for experts because they already have knowledge of protein shapes. Another noted the abstract is enough unless there is a need to learn about specific binding sites on the proteins for a reaction to occur.

For understanding the structures, movement, and spatial understanding, the 3D models used in the videos were considered more beneficial (n = 9). When deciding between seeing the continuous process and then revisiting content (Narrated Video and Video) compared to the overview layout with dynamic components (Hybrid), the experts preferred continuous video (n = 5). At the same time, one noted that the video ought to have segmenting elements that signal key steps. The overview using dynamic steps (Hybrid) was preferred by three experts.

#### 5.3.3 Purpose of representations

Table 3 summarises how the experts would use the representations in real life. They were asked to give open answers, which we categorized into three main categories: education, printed media, and public engagement in accordance with thematic analysis. The education category involved responses such as graduate teaching and lectures. The printed media encompassed posters, journal publications, supplementary materials, and textbook content. The public engagement category included science centers, entertainment, and advertisements. The Narrated Video and Detailed Static representations were seen as the most useful in education (n = 6). The Abstract Static representation was most often suggested for printed media (n = 5). For dissemination, Narrated Video was seen as the most suitable option (n = 8).

**Table 3:** A summary of the use of each of the representations divided into three categories; education, public relations, and printed media.

	Education	Printed media	Public
**Detailed Static**	6	3	2
**Abstract Static**	3	5	3
**Hybrid**	5	1	2
**Narrated Video**	6	0	8
**Video**	4	0	3

### 5.4 Focus group

The focus group provided insight into each of the different representations. Overall, the experts did not have a straightforward answer about which representation they find the most and least useful for teaching high school students about the biochemical processes. They discussed attributes of each representation, such as their ability to convey movement, sequence, and visual appearance. During the discussion, the experts seemed to naturally divide general comments between the representations with an overview approach showing all the steps of the process (Detailed Static, Abstract Static, and Hybrid) versus a continuous communication of the process (Video and Narrated Video).

#### 5.4.1 Impressions

The presented five representations were considered very good and were cherry-picked as the best from many more available. So the difference between them may be so small that it does not even matter in the grand scheme of effective learning. In this section, we discuss comments on the visual appearance, layout, motion and function communication, and other remarks.

The Abstract Static representation gave metaphors for function, specifically, a metaphor for mechanical car engines, which can be used to explain the energy-making process in the cellular mitochondria. This has the potential as an effective storytelling technique. It was noted that the smaller actors in the representations, such as the small hydrogen molecules, were lost because the enzymes were considered the main components and took the attention. In terms of their visual appeal, they were also described as having ‘too many edges’ and considered unappealing. The expert with a main background in design explained that humans observing biological processes want to look inside a living cell; hence the depictions should show an organic-looking environment. Another expert preferred realistic-looking depictions rather than ‘cartoon-like’ illustrations of molecules. From these two opposing comments, it is evident that the apparent preference of presented data varies between people and their domains. The level of detail was challenged as unnecessary when a lot of movement was shown in the continuous video representations. However, the level of detail and its organic aesthetics were also considered good for inspiring students to want to learn about biochemical processes. All the experts concluded that the videos were very appealing but contained too much information and visual noise, which would not be compartmentalized immediately. No meaningful learning would concur, despite catching attention. Another interesting point about visuals related to Abstract Static representations was an observation that school curricula do not encourage abstract thinking. For example, the more realistically the pieces are rendered in art, the more ‘correct’ they are considered. This could be one of the explanations for not favoring the Abstract Static representation.

The Detailed Static representation was seen as suitable for understanding general movement due to the use of arrows and numbers indicating individual steps of the process. For sequence, the experts commented that it is clearly understandable in the overview sequential representations, such as the Static and Hybrid representations. This was a result of the numbering of the steps. The numbering also suggests the order of events, however, it was mentioned that this could be misleading because all these steps are happening simultaneously. Also, the viewer may assume that when part of the process is shown earlier than another, it means that it is more important, which may not always be the case. The video representations were considered suitable to be showing sequence and all the processes going on simultaneously. However, it was noted that students need to exert more cognitive effort to identify the important steps in the biomolecular process in the continuous presentation.

The benefits of the videos were also repetitive movements of the ongoing processes, which would help with understanding certain parts of the processes, despite looking abstract. The video representations were labeled as showing very fine-grained detail of movement. In this case, the question was raised whether that level of detail is needed. Unsurprisingly, all the videos used were considered beneficial in showing dynamic processes.

#### 5.4.2 Purpose of each representation

Different representations were suggested for different uses to be the most effective learning aid for specific target groups and media. The Detailed Static representation was labeled as a useful tool for revising for an exam. It was also mentioned that because of its size, the presentation of this medium, on posters or in textbooks, should be larger than the Abstract Static representation with fewer details. The Abstract Static representation could occupy a smaller space and be accompanied by more text. The illustrations were also called pretty at one point, and they would look nice on t-shirts, which could be incorporated into public engagement. The Hybrid representation could be used for teaching at school if it was divided into physical stations. Each station would represent a stage in the molecular process and be explained in detail with the use of, for example, tablets. The physical division and movement between stations would make the process more memorable. It was also suggested that there could be a combination of static media and Hybrid representation by creating an interactive poster with Augmented Reality. However, experts in the educational domain stated that students do not always find novelty media, such as virtual or augmented reality, more attractive. It is more suitable for children with more energy to interact with that kind of media. The Video without Commentary was considered a good option for lectures and conferences, where the speaker could narrate the video live. A statement opposed to this is that it makes the Video similar to Narrated Video. However, live interaction differs from passively watching Narrated Video. The Video and Narrated Video were considered helpful in public engagement, such as museum presentations. The Narrated Video was also considered very useful for teaching.

#### 5.4.3 Suggested new versions

In addition to some previously mentioned alterations to the existing representations and critiques, the experts suggested new versions of the ATP synthesis process. This included a comic strip and illustrations with an isometric view to show the depth of field. Comics have been proven to be useful in data science before [33, 38]. The isometric view would be beneficial to showing a third dimension on a 2D surface.

The second suggestion was 3D printed physical models that could be taken apart (and were considered prior to this study, but due to the inability to test 3D physical models online, this representation was not made). The use of tangible models would also be useful as previous studies into physicalizations of medical data were appropriate for educational visualizations [50, 51].

It was also suggested to make a cartoon video in the style of the two static representations. This approach has the potential to simplify the visual clutter that some participants mentioned and simplify the chemical actors.

Dynamic representation could be made with 3D models showing the same process but from several different points of view and could incorporate arrows and texts. As mentioned before, the 3D models would help understand molecular interactions [21], dynamic and spatial interactions [23], and the different views [24] on the same process and actors would provide an even higher degree of spatial organization.

Two opposing ideas were presented: one by the educational expert who suggested that children could be presented with a representation that shows all the steps in the process and has no dynamic components to understand the process and then be presented with a Narrated Video to fill in the gaps. On the other hand, an expert in bio-visualization suggested a reversed approach where the whole process is shown, and then the viewer goes into a detailed study of each step. The combination of static and dynamic aspects would have the same advantages that we meant for the Hybrid one, but starting with a simpler representation might be better for grasping the basic concept and only then adding the more complex details, such as in animation.

Another idea was to create a video with labeled timestamps marking each critical step, such as educational videos on the YouTube platform. Another suggestion was to show slower videos in the native language of the viewers. Slowing down was probably suggested because the video was thought to be quite fast-paced and complex to grasp all the details (a similar reason why the combination was suggested).

A hybrid version could also be presented on a scrolling website with an overview of the entire process in the corner of the screen.

Another great suggestion was to let the students hand-draw the process after watching a video to create their own learning material by creating a concept from detailed visuals and abstracting it in a suitable way.

It was also said that technology makes teaching a lot easier nowadays because many of these options were not available a few years ago; in the next ten years, new technology will be available to be utilized in education. Overall, the representations that are easier to retrieve are considered to be more effective as teaching tools.

#### 5.4.4 Comments on results of non-experts and experts

The discussion about the results from the previous groups allowed us to find possible explanations for why the participants from the two target groups found the representations helpful or unhelpful. The opposing preferences and dislikes of experts and non-experts were unexpected. One of the possible explanations was the fast speed of the video as discussed previously. It was also suggested that the new biology-specific terms not available in written form might have negatively affected the understanding of the Narrated Video by non-experts since it would be more challenging to search and understand those terms in the video. This was also indicated in their open feedback comments. Another possible explanation for this was the language barrier since many of the participants were not native English speakers. Therefore, we carried out post-hoc tests. We have determined that the language barrier was not a reason for participants’ lower scores in the non-expert group because participants with different levels of English language knowledge (including native speakers) had similar results. The Narrated Video with the lowest score and the Detailed Static group with the highest score both had participants from English-speaking countries.

## 6 Discussion

In this section, we discuss the findings from the extensive study, including lessons learned from open feedback from all three groups and recommendations for improvement for each representation.

### 6.1 Lessons leaned

The results indicate that different target groups prefer different representations. We identified causes for these preferences, such as complexity, level of detail, familiarity with the concept, and ease of finding information. The familiarity and past experience with interpreting visual scientific representations can also affect the level of competence in interpreting them. As such, the experts with vast previous experience are better at deciphering and using representations and are more likely to gravitate towards more complex choices.

Overall the collected data indicated that the non-experts prefer to view the biochemical processes to be represented in an overview scheme showing the main phases of the process, unlike experts who prefer continuous representations. This approach was the second favorite of the expert group, just after the Narrated Video. This contradicted the non-expert group, which deemed the Narrated Video the least helpful and chaotic. We have concluded that different representations have different uses for different groups. The student groups’ qualitative and quantitative results indicate that a static overview is the most beneficial as it allows going back to the desired part of the process. This is a claim that was also supported by some of the experts in the focus group.

Our assumption was that a combination of static overview with dynamic videos in the form of Hybrid representation would be the most successful and favored by both groups, non-experts, and experts. This was because it provides a large amount of detail and insight into the order of events, movement, quick overview, recollection, and auditory effects combined with the overview and segmenting approach [6]. In the case of the Hybrid, the non-experts also prefer static representation in overview scheme rather than with dynamic components. Meanwhile, experts prefer the dynamic Hybrid components in the overview scheme. New biology-specific terms, as indicated by non-expert responses, are also the reason why combinations of different media, including written text, are necessary. Most of the scientific education material will introduce unknown terminology and thus would face the same problem as using just narrated video content.

Our results agree with the claims that representations must contain information but not be too abstracted or too detailed [31]. On the other hand, a participant in the focus group stated that he would expect experts to be able to use simple diagrams as they already possess the knowledge of simple representations. Another expert stated that these were the only representations of unseen abstract proteins before technology allowed us to visualize them. However, the results of our study show that non-experts get overloaded with visuals and content information and perform worse on the content questions. We also agree that the point of representations is to create effective communication rather than focus on aesthetics [31]. We assume that the more knowledge people have about a topic, the more information they can perceive without being overloaded. The Theory of Multimedia Learning [15] explains why some of the representations were not as effective; for example, the Narrated Video was too detailed while a lot of movement was happening on screen. The non-expert and expert groups both preferred detailed representations compared to abstract representations. However, the experts from the focus group suggested its use in printed media and use for people who already have knowledge in that area.

The keywords that were selected in the non-expert and expert surveys helped us to understand how each group perceives the visualizations. This is important as attractive visualizations that are eye-catching and stimulating prompt learning [52]. Considering attractiveness in multimedia, for example, how ‘pretty’ or ‘busy’ they appear, play an important role in learning motivation. For example, visually appealing images prompt people to learn more [53]. Similarly, attractive and effective visual representations in an expert context, such as use in visual abstracts, play a role in the perception of the research they represent and how reliable the researchers are perceived. For example, a properly visualized visual abstract helps the manuscript to be rated as well written and researchers to be considered more reliable [54]. Visual abstracts are becoming more popular in science, which also influences the rate of their dissemination, for example, through social media popular in the scientific research community [55]. In the end, the impressions the scientific visuals make are important in expert and non-expert contexts, and hence it is necessary to pinpoint what impression they make on each group.

One aspect that hinders the learning of the ATP process specifically is the lack of explicit connection between chemistry and biology information [12]. We believe that the representation in the preferred non-expert form would be most beneficial for students at the high school level to learn about it as it explains chemistry applied to biology visually but without overwhelming complexity. Further, we argue that appropriate visual representation can make a clear connection between these two disciplines, especially in helping explain the APT synthesis [13] and similar processes [14].

### 6.2 Recommendations

Overall, with the results from the qualitative and quantitative data collected, we came to a conclusion about each of the representations and listed our recommendations for their use. The high results of Detailed Static representation quantitatively combined with the repeated comments appreciating the step-by-step approach and detail make it a very effective learning tool for high-school students learning for exams. It would be even more beneficial to use it in combination with the Narrated Video, despite the non-experts’ low scores and qualitative critique because once they get the basic idea of the actors and processes from overview, they can focus on understanding the continuity of the process through dynamic continuous video with narration. The Abstract Static is also beneficial in education, however, based on the results from all the groups, it would be most helpful in printed media like journals and higher-level textbooks targeted at experts. We recommend abstracted illustrations to be used where readers are looking to add the knowledge they already possess and do not need to be taught basics. Details should be used to focus on the new information. We would also like to point to the comments that were left by non-experts and that some have enjoyed and some disliked the color palettes in the illustrations; we agree that certain rules about color palettes in professional molecular visualizations should be followed to achieve harmony [56]. This was also suggested in the focus group by an expert who remarked that even the colors shown in representations of processes are abstractions since we cannot see the proteins in real life.

We anticipate that the Hybrid representation would be most helpful in the education of high school students, where each of the steps could be made into a detailed and interactive activity at different physical stations representing each step. This type of interactivity would also make it suitable for science museums where people are willing to put effort into interaction for the sake of learning. We suggest that the Narrated Video would be beneficial for both learning in the classroom as well as teaching the public and getting people interested in science. However, an audience-appropriate voice-over would need to be used for each specific target group to not overwhelm the novices or not under-stimulate experts who would like to point out new, detailed findings that require prior knowledge. Based on the feedback, we also think that the attractive graphic would help attract students and novices to science, as well as reinforce the experts’ belief that science is beautiful and interesting. For both groups, we also suggest the narrated videos should have the option to be paused as well as have markers pointing to the critical points in the process for easier navigation. The Video ought to be used in combination with live narration based on the audience’s level of understanding. The live interactivity would allow the viewers to ask questions at the moment. Its interactivity would therefore be channeled through the speaker, who would manipulate the video based on the audience’s reactions.

### 6.3 Validity

The external validity of this study is high as more biochemical processes and other abstract occurrences with time sequences could be studied similarly. Internal validity is high as we employed two study iterations with different populations of experts and non-experts. The study also employed comparisons between individually answered questionnaires (non-experts), people who compared all five versions with each other, and in the end, a focus group, where participants viewed all the representations and were familiarised with the results of the previous test groups and got to discuss them. Ecological validity is high as we asked participants who use and create visualizations, study, and work with biomedical data to participate in our study. In addition, the representation versions were also based on a professionally done biochemical visualization which is considered accurate from the scientific point of view and the point of view of an educational tool.

### 6.4 Limitations

It would be more accommodating if the student population were between 120 to 140 participants. While we had a representation of different backgrounds in each representation for non-expert iteration, we would benefit from having equal representation of biochemical, computer science, and other backgrounds in each representation group. However, the overall number of participants was large enough to generalize the findings to other populations within STEM subjects and also included people from international backgrounds.

We acknowledge that experts might have been subjected to Order Effect [57] as the representations displayed were always of the same process. One participant mentioned that it is possible that he understood the biochemical process because he had seen all the other representations before the Narrated Video (last in order), which gave him the knowledge, and that was why he deemed it most useful. However, the said participant had over 30 years of experience with biology, so the order of events did not seem relevant in his particular case.

Another critique of our study design could be the conflation of variables, such as using static versus dynamic, abstract versus detailed, and overview versus continuous approaches mixed within our representations. However, in reality, those aspects are used in combinations and not as isolated concepts. Furthermore, it was shown [15] (and confirmed also by our study) that combining different representation aspects that are beneficial by themselves does not necessarily yield better outcomes. Thus evaluating the combinations is necessary. We revised the related work and carefully designed our representations based on the known or expected benefits of their various aspects. We compared not only already well-studied representations against each other, such as overview storyboards versus dynamic videos, but we have also designed a novel Hybrid representation that incorporated the benefits of the two approaches. Therefore, the use of the different representations with different variables was a deliberate choice and part of our study design. When we consider simulating the learning process, which we also measured by enquiring how many times the participants have revisited the representations, we consider that we have succeeded as all except 5 participants have revisited the material several times. People who have revisited the material several times but did not constantly go back were simulating learning for an exam. They were focused enough to not constantly check for answers (as the 5 participants who went back 10+ times) but also did not skim it only once like the 4 participants who did not go back at all. However, we do acknowledge that learners learning for exams would make learning a lot more serious where failure on tests has academic consequences, compared to anonymous people who are part of a survey.

An aspect to consider may be that since the participants could revisit the representations after reading the questions, the study possibly measured how well the participants could find the answers, rather than how well the participants learned about the process. However, we deliberately let the participants return to the material and asked them to indicate how many times they did so to simulate studying for an exam. In a real-life setting, students would go through the material several times instead of just looking at it once.

To mitigate the limitations of our study set-up, we designed our survey question to also test for the understanding of what is being shown rather than just for recollection. A suggestion for future setup to study learning versus memorization would be to measure participants’ response times to questions revealed to them before they watch the representations to know what they should focus on.

### 6.5 Future work

As we have discussed, one of the reasons leading to the different representation preferences between the target groups could be their level of competence in interpreting complex representations. A future study could thus focus on investigating and evaluating methods and resources that the experts (i.e., educators) are currently using to help their students understand and decipher different representations, such as the examples used in our survey.

Another interesting avenue to explore would be the application of different representations on different scales of biochemistry. For example, leaving the micro level with the biochemical processes and traversing across to the larger scale with, for example, blood flow or even the processes on the meso scale with human anatomy of organs. In the future, we would like to test approaches indicated by the focus group, such as 3D printed physical models and a combination of several different representations, such as video and static illustration. It would be interesting to take the concept of data comics [4] and compare the effectiveness of visualization between biochemical processes depicted as comic strips in realistic depiction and depiction as metaphors, as suggested by the focus group and discussed by Alemany-Pages [35]. The study design would ideally involve classroom testing when the different representations would be used in practice and integrated into the curriculum.

## 7 Conclusion

In this paper, we have focused on media of illustration and video animation as they are easily accessible, despite more technology, such as Virtual or Augmented Realities. We have assessed their effectiveness in learning by testing them individually and creating a version that combines them. We have answered the questions posed at the beginning of our research.

We suggest the combination of different representations would be the best approach to learning about biochemical processes, such as being presented with a simple overview first and then watching a video, rather than combining those two approaches, as suggested by experts and the focus group.

Based on the results from the expert group, we can conclude that representations containing a lot of details and information are suitable for people with background knowledge in the subject. Unsurprisingly, movement and space are best understood with 3D models.

Our study has shown unexpected results that we would like to explore in the future using further study into creating new materials for testing the Hybrid visualization and branching out into different levels of abstraction in the static overview representations.

## Supporting information

S1 File Representations. File containing a link to the website with representations and screenshots of the individual representations.
(PDF)

S2 File Questionnaires. File containing questionnaires for two test groups and an itinerary for the focus group.
(ZIP)

S3 File Non-expert participants. File containing the non-expert demographics and group division.
(PDF)

S4 File Expert participants. File containing the expert demographics and group division.
(PDF)

S5 File Focus group participants. File containing the demographics description of the experts participating in the Focus Group.
(PDF)
