# Supplementary material for: Seeing the unseen: Comparison study of representation approaches for biochemical processes in education
Source: PLoS One. 2023 Nov 6;18(11):e0293592. doi: 10.1371/journal.pone.0293592 (PMC10627439; doi:10.1371/journal.pone.0293592)

S1 Supplementary Materials: File containing a link to the website with representations and screenshots of the individual representations.

Link: https://decibel.fi.muni.cz/study/atp_synthesis/

Homepage:

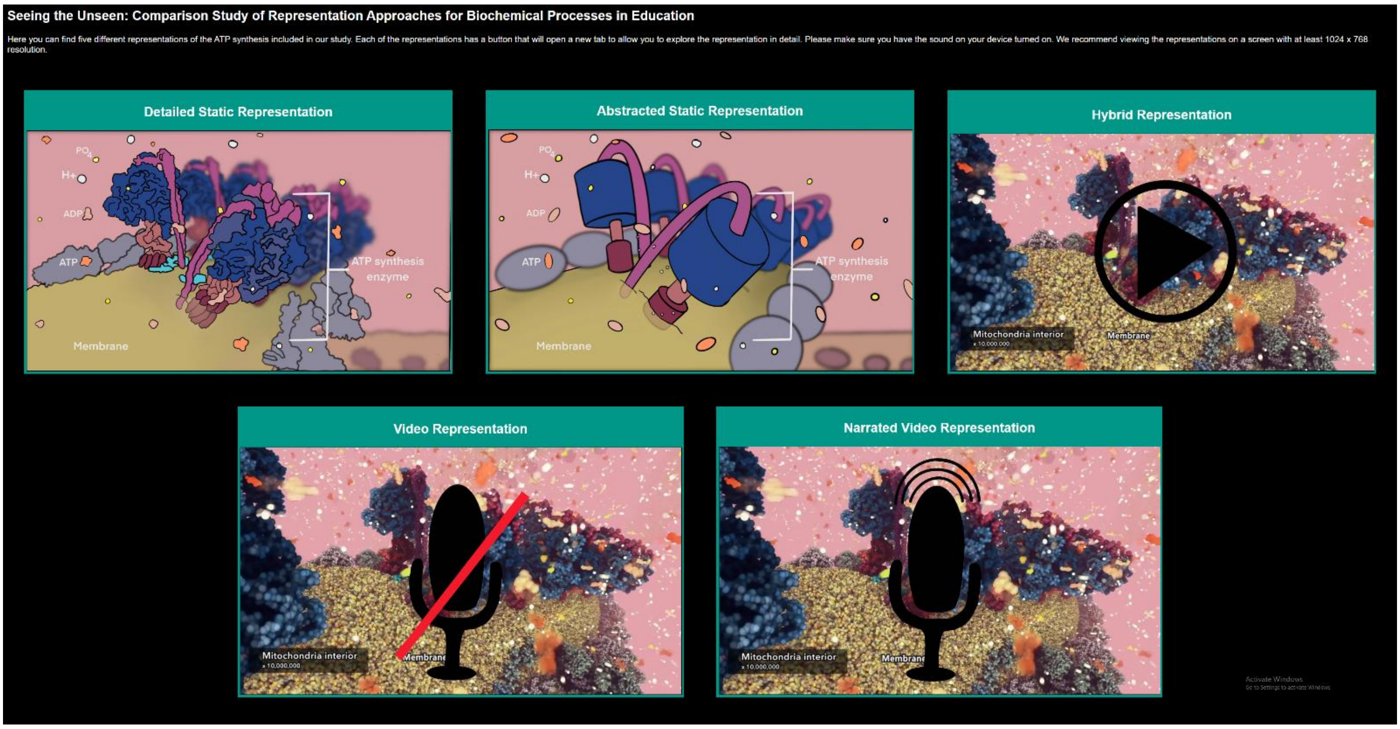

Detailed Static Representation

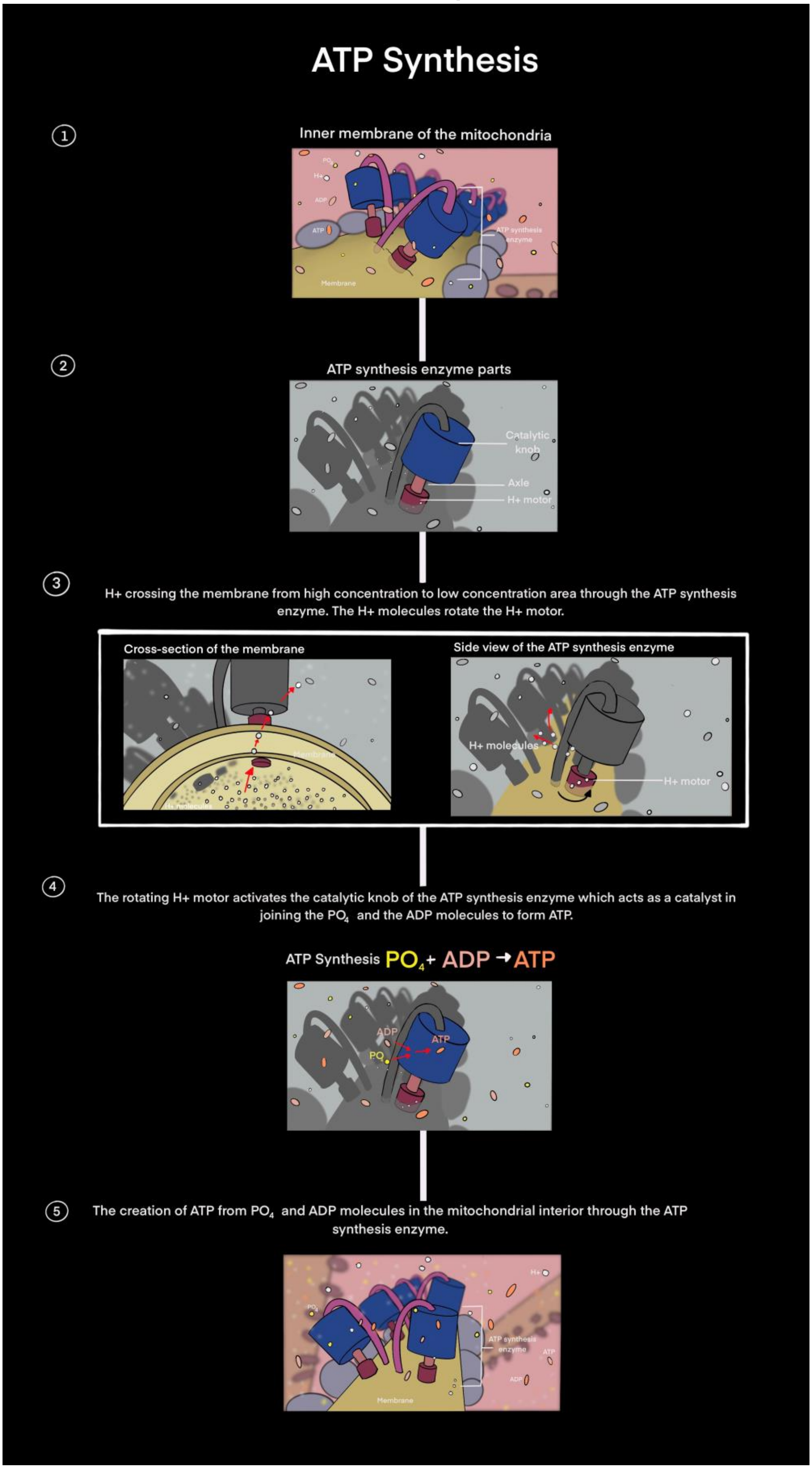

Abstract Static Representation

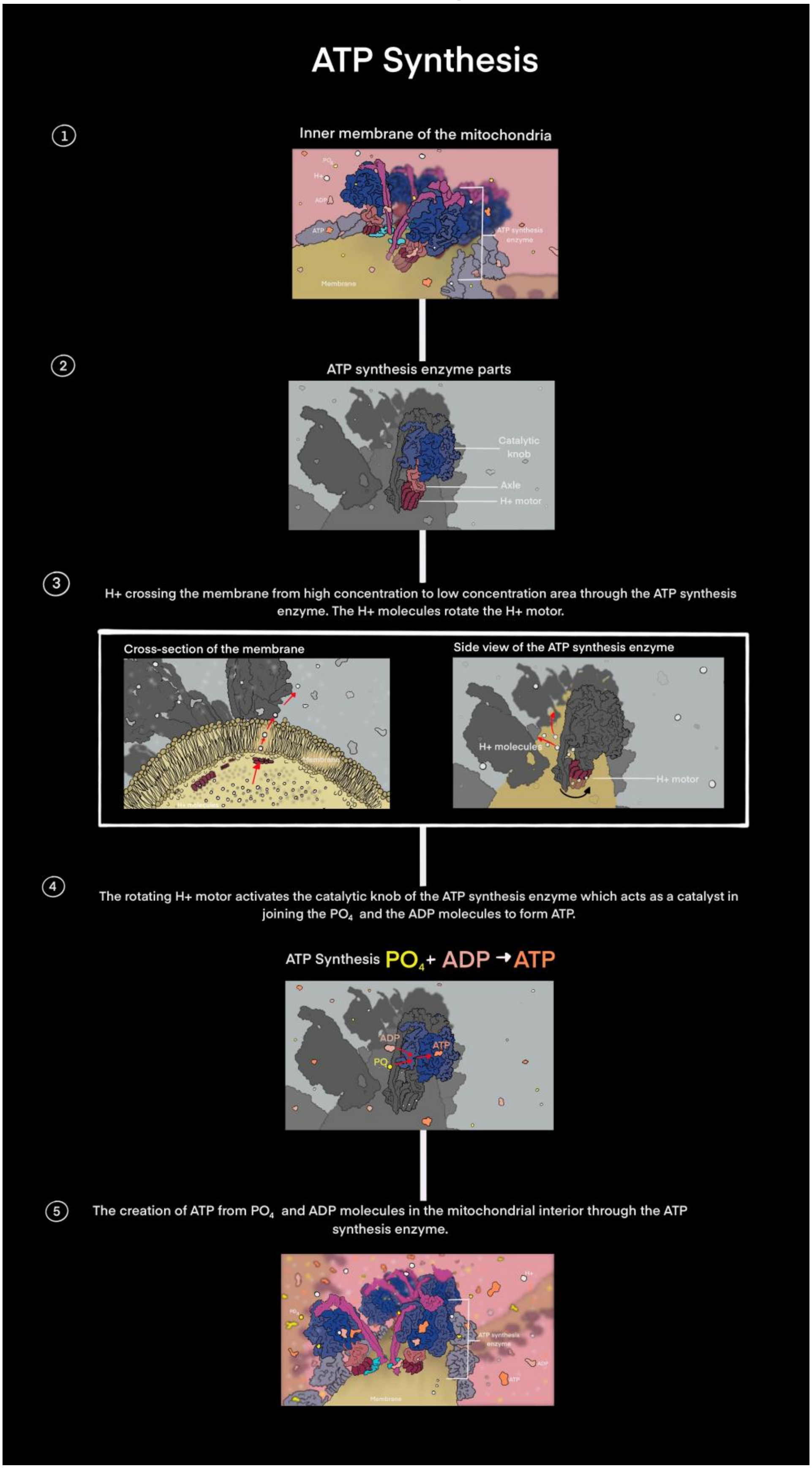

Hybrid Representation

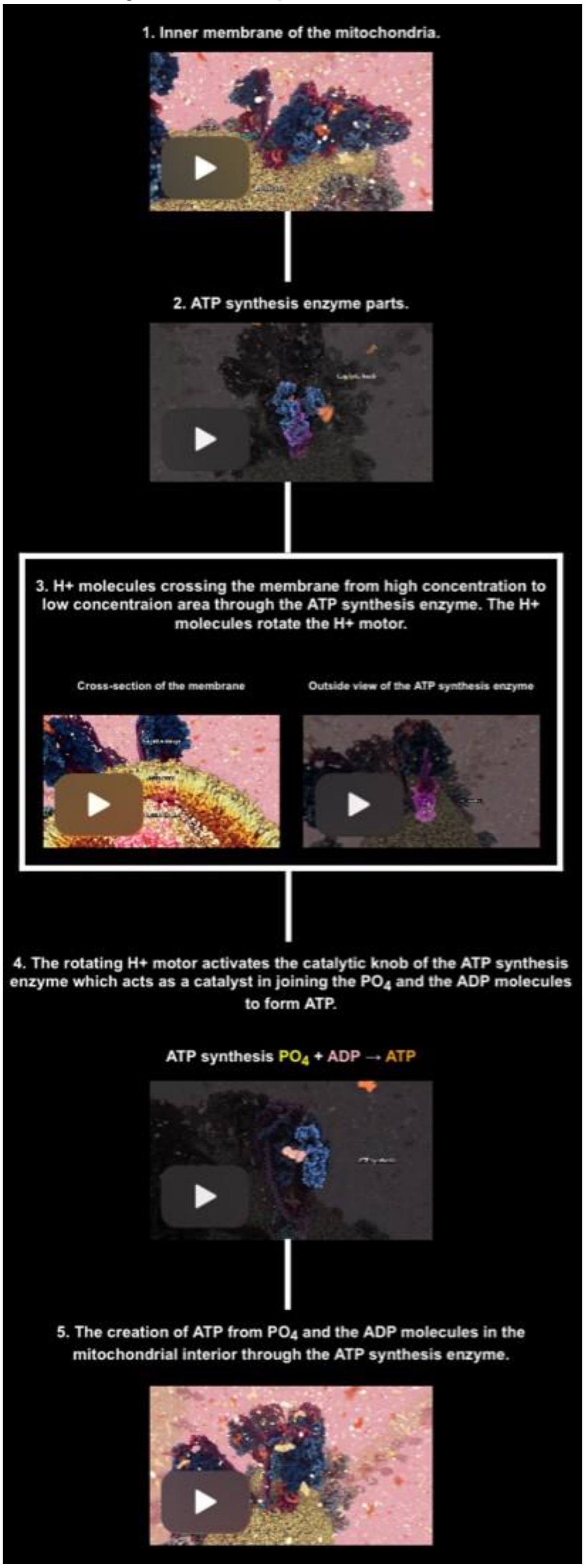

Video Representation

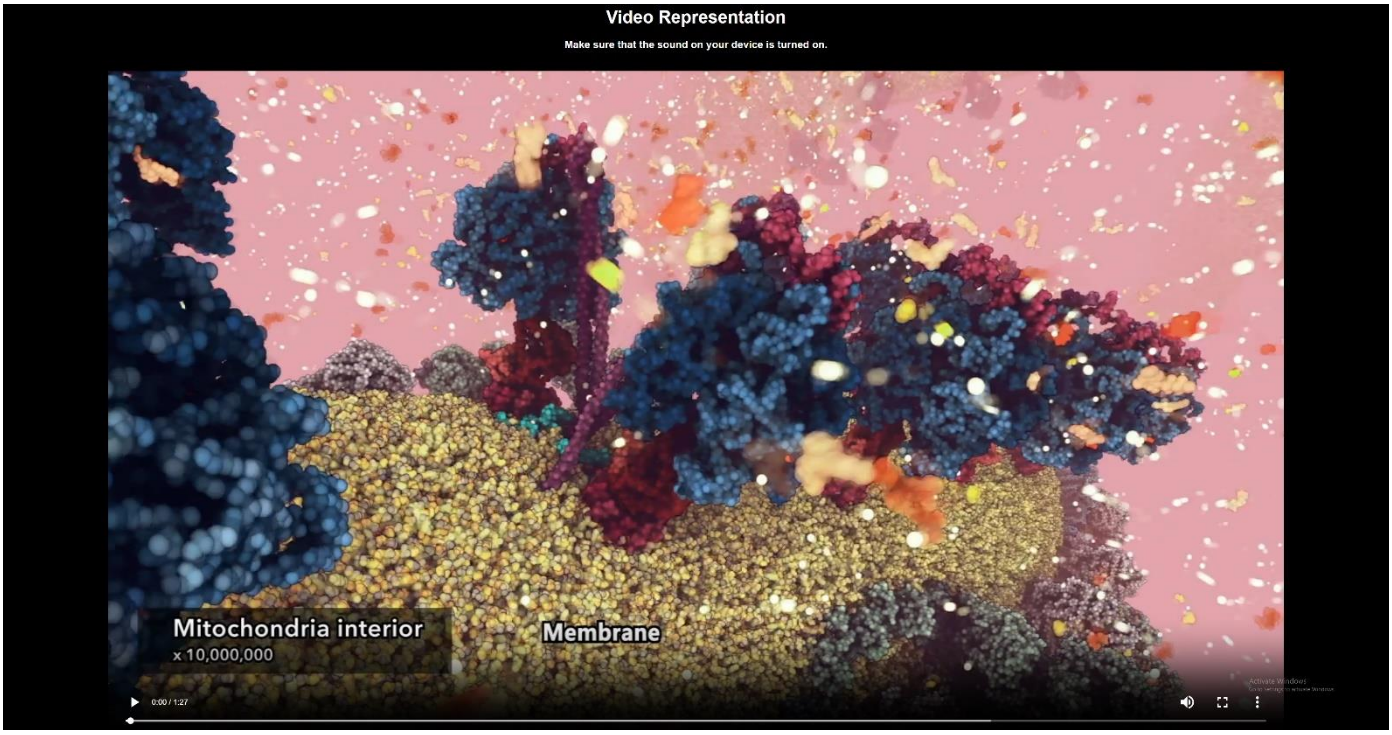

Narrated Video Representation

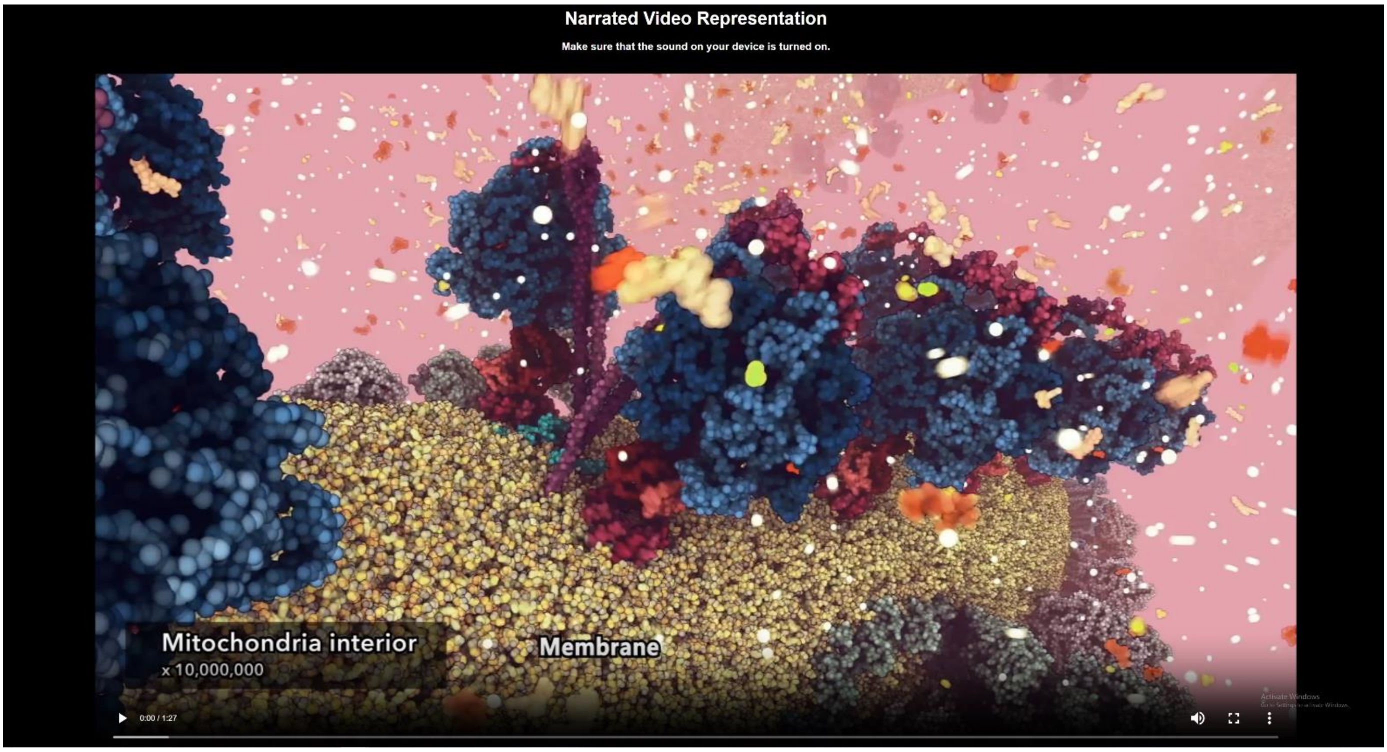

Supplement: S1 File — File containing a link to the website with representations and screenshots of the individual representations. (PDF) [file pone.0293592.s001.pdf]
