# Supplementary material for: Seeing the unseen: Comparison study of representation approaches for biochemical processes in education
Source: PLoS One. 2023 Nov 6;18(11):e0293592. doi: 10.1371/journal.pone.0293592 (PMC10627439; doi:10.1371/journal.pone.0293592)
Supplement: S2 File — File containing questionnaires for two test groups and an itinerary for the focus group. (ZIP) [file pone.0293592.s002.zip › Questionnaires/Itinerary and Discussion Questions_FOCUS_GROUP.pdf]

Itinerary and Discussion Questions: Focus Group

1. Introduction of everyone
2. Filling in consent
3. Representations in detail
4. Part 1 - general questions about the representations
   - Which representation is the **most helpful** in understanding the process based on your field of expertise, and why?
   - Which representation was the **least helpful** in understanding the process based on your field of expertise, and why?
5. Break
6. Part 2 - discussing each representation individually
   - How helpful is the representation in understanding the **movement** of molecules and enzyme?
   - How helpful is the representation in understanding the **sequence of events** in the process of ATP synthesis?
   - Could you comment on the **visual appeal** of the representation?
   - For which **purpose** do you think this representation is most suitable?

     Do you have any suggestions for improvements or changes?
   - Would you propose different representations?
7. Part 3 - presenting and discussing the results of the user studies with students and experts
   - Based on the presented results of surveys, did you change your opinions that we have previously discussed?
   - Do you have any further comments or suggestions?
8. Debrief and conclusion
