# Supplementary material for: Seeing the unseen: Comparison study of representation approaches for biochemical processes in education
Source: PLoS One. 2023 Nov 6;18(11):e0293592. doi: 10.1371/journal.pone.0293592 (PMC10627439; doi:10.1371/journal.pone.0293592)
Supplement: S2 File — File containing questionnaires for two test groups and an itinerary for the focus group. (ZIP) [file pone.0293592.s002.zip › Questionnaires/Online Survey Questionnaire_EXPERTS.pdf]

Online Survey Questionnaire: Experts

Preferences

---

1. Which representation was the **most** helpful in understanding the process, and why?

- ○ Detailed Static
- ○ Abstracted Static
- ○ Hybrid
- ○ Video
- ○ Narrated Video

Why is this modality most helpful and why?

---

2. Which representation was the **least** helpful in understanding the process, and why?

- ○ Detailed Static
- ○ Abstracted Static
- ○ Hybrid
- ○ Video
- ○ Narrated Video

Why is this modality least helpful and why?

---

3. Choose options that best describe the **Detailed Static Representation**:

- ☐ Simplistic
- ☐ Clear
- ☐ Detailed
- ☐ Excessive
- ☐ Informative
- ☐ Confusing
- ☐ Pretty
- ☐ Misleading
- ☐ Distracting
- ☐ Easy to read

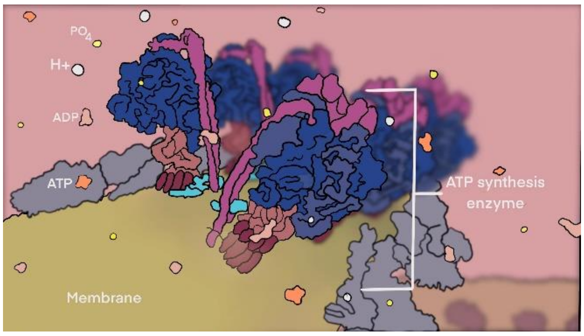

☐ Inaccurate

☐ Precise

☐ Accurate

☐ Visually [illegible] Please specify here

☐ Other

---

4. Is there something you would change in this representation to show the process in a more understandable way?

Enter your answer here

---

5. Choose options that best describe the **Abstracted Static Representation**:

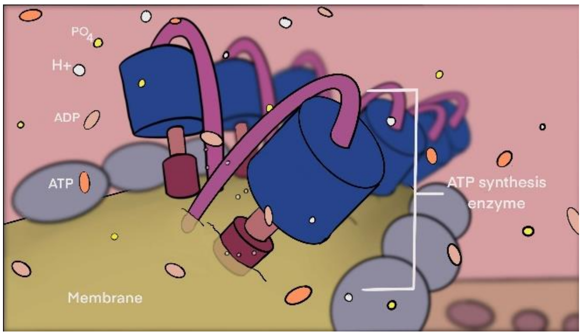

☐ Simplistic

☐ Clear

☐ Detailed

☐ Excessive

☐ Informative

☐ Confusing

☐ Pretty

☐ Misleading

☐ Distracting

☐ Easy to read

☐ Inaccurate

☐ Precise

☐ Accurate

☐ Visually unappealing

☐ Other Please specify here

---

6. Is there something you would change in this representation to show the process in a more understandable way?

Enter your answer here

---

7. Choose options that best describe the **Hybrid Representation**:

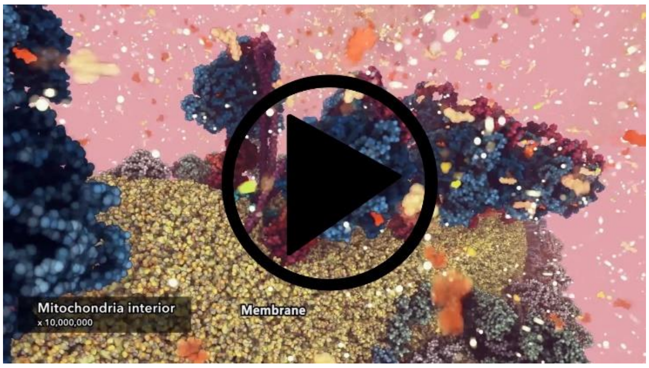

- ☐ Simplistic
- ☐ Clear
- ☐ Detailed
- ☐ Excessive
- ☐ Informative
- ☐ Confusing
- ☐ Pretty
- ☐ Misleading
- ☐ Distracting
- ☐ Easy to read
- ☐ Inaccurate
- ☐ Precise
- ☐ Accurate
- ☐ Visually unappealing
- ☐ Other [Please specify here]

---

8. Is there something you would change in this representation to show the process in a more understandable way?

[Enter your answer here]

---

9. Choose options that best describe the **Video Representation**:

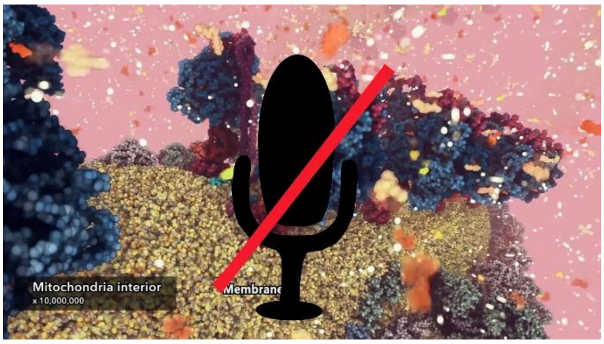

- ☐ Simplistic
- ☐ Clear
- ☐ Detailed
- ☐ Excessive
- ☐ Informative
- ☐ Confusing
- ☐ Pretty
- ☐ Misleading
- ☐ Distracting
- ☐ Easy to read

- [ ] Inaccurate
- [ ] Precise
- [ ] Accurate
- [ ] Visually unappealing
- [ ] Other [Please specify here]

10. Is there something you would change in this representation to show the process in a more understandable way?

[Enter your answer here]

11. Choose options that best describe the **Narrated Video Representation**:

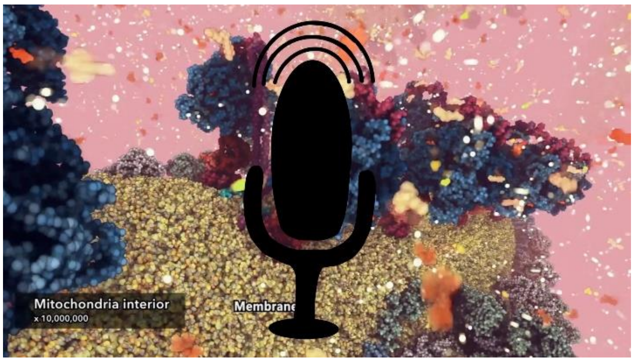

- [ ] Simplistic
- [ ] Clear
- [ ] Detailed
- [ ] Excessive
- [ ] Informative
- [ ] Confusing
- [ ] Pretty
- [ ] Misleading
- [ ] Distracting
- [ ] Easy to read
- [ ] Inaccurate
- [ ] Precise
- [ ] Accurate
- [ ] Visually unappealing
- [ ] Other [Please specify here]

12. Is there something you would change in this representation to show the process in a more understandable way?

[Enter your answer here]

Movement and sequential data

13. Which representation was the most helpful in understanding the **movement of molecules and enzyme**?

- Detailed Static
- Abstracted Static
- Hybrid
- Video
- Narrated Video

---

14. Based on your previous answer, what helped you understand the movement of the molecules?

Enter your answer here

---

15. Which representation was the most helpful in understanding the **sequence of events** in the process of ATP synthesis?

- Detailed Static
- Abstracted Static
- Hybrid
- Video
- Narrated Video

---

16. Based on your previous answer, what helped you understand the sequence of events in the ATP synthesis process?

Enter your answer here

Learning and comparisons

17. For understanding of the process, is it more helpful to see the overview of the entire process (Detailed Static, Abstracted Static and Hybrid Representations) or in a continuous video (Video and Narrated Video Representations)? And why?

Enter your answer here

---

18. Is the Abstracted Static Representation sufficient for correct understanding of the process? Or do you prefer the Detailed Static Representation?

Enter your answer here

---

19. Are the 3D models in the Hybrid and animated representations (Video and Narrated Video Representations) helpful in understanding the movement and spatial relationships? Or are the static representations (Detailed Static and Abstracted Static Representations) sufficient?

Enter your answer here

---

20. In the animated representations (Hybrid, Video and Narrated Video Representations), do you prefer to see the process as a continuous video (Video and Narrated Video Representations) where you can watch and revise the process or do you prefer to watch each step of the process separately (Hybrid Representation)?

Enter your answer here

---

21. In which domains for which tasks would each of the five representations be most useful? For example education, dissemination, entertainment... You can write more than one task for each representation.

- Detailed Static Enter your answer here
- Abstracted Static Enter your answer here
- Hybrid Enter your answer here
- Video Enter your answer here
- Narrated Video Enter your answer here

---

22. Please leave any other comments about the representations here:

Enter your answer here
