# Supplementary material for: Seeing the unseen: Comparison study of representation approaches for biochemical processes in education
Source: PLoS One. 2023 Nov 6;18(11):e0293592. doi: 10.1371/journal.pone.0293592 (PMC10627439; doi:10.1371/journal.pone.0293592)
Supplement: S2 File — File containing questionnaires for two test groups and an itinerary for the focus group. (ZIP) [file pone.0293592.s002.zip › Questionnaires/Online Survey Questionnaire_NON_EXPERTS.pdf]

Online Survey Questionnaire: Non-Experts

1. In which part of the enzyme do the molecules fuse into ATP?

Enter your answer here

2. Which two molecules react to form the ATP molecule?

Enter your answer here

3. Which part of the cell does the ATP synthesis occur in?

Enter your answer here

4. What role do the H+ molecules play in ATP synthesis?

Enter your answer here

5. How do the H+ molecules cross the membrane?

Enter your answer here

6. Which side of the membrane does the ATP synthesis reaction occur?

Enter your answer here

7. Put the following steps of the ATP synthesis process in the correct order by dragging and dropping.

- Step 1
- Step 2
- Step 3
- Step 4

ATP release

H+ motor activation

ADP and PO_4 reaction

H+ crossing

8. Choose the options that best describe the representation of the ATP synthesis process. You can choose several options:

- [ ] Simplistic
- [ ] Clear
- [ ] Detailed
- [ ] Excessive
- [ ] Informative
- [ ] Confusing
- [ ] Pretty
- [ ] Misleading
- [ ] Distracting
- [ ] Easy to read
- [ ] Inaccurate
- [ ] Precise
- [ ] Accurate
- [ ] Visually unappealing
- [ ] Other [Please specify here]

---

9. In your own words, write at least three things that you liked the most about the representation:

[Enter your answer here]

---

10. In your own words, write at least three things that you did not like about the representation:

[Enter your answer here]

---

11. Please indicate on the scale below how well the representation communicated the order of events in the ATP synthesis process. Number 1 indicates that the representation did not communicate the order of events in the process clearly at all, while number 5 indicates that the order of events was communicated very clearly.

- ( ) 1: The order of events was not represented clearly at all.
- ( ) 2
- ( ) 3
- ( ) 4
- ( ) 5: The order of events was represented very clearly.

---

12. Please indicate on the scale bellow how well you understood the movements happening within the ATP synthesis process. Number 1 indicates that you have not understood the movements in the ATP synthesis process at all, while number 5 indicates that you understood the movements very well.

- 1: I did not understand the movements at all.
- 2
- 3
- 4
- 5: I understood the movements very well.

---

13. How often did you look back at the representation to answer the follow-up questions?

- Never
- 1-5 times
- 6-10 times
- More than ten times

---

14. Please leave any other comments about the representation:

Enter your answer here
