# Supplementary material for: Seeing the unseen: Comparison study of representation approaches for biochemical processes in education
Source: PLoS One. 2023 Nov 6;18(11):e0293592. doi: 10.1371/journal.pone.0293592 (PMC10627439; doi:10.1371/journal.pone.0293592)
Supplement: S3 File — File containing the non-expert demographics and group division. (PDF) [file pone.0293592.s003.pdf]

S3 Supplementary Materials: Non-expert demographics and group division

**Detailed Static** (n=14)					
Gender	Age	Field of study/work	Biology Experience	Visualization Experience	Vision
Male	21	Visual Informatics	High school	Consumer, Producer	mild deutan
Male	20	Programming and application development	High school	Cpnsumer, Producer	Normal
Male	23	Computer games and Graphics	High school	Consumer, Producer	Normal
Female	20	Economics	Hobby	Consumer	Normal
Female	24	Digital Forensics	Expert	Producer	Normal
Female	20	Multimedia	Not interested	Not using	Normal
Female	21	Computer graphics	High school	Consumer	Normal
Female	20	Molecular biology and genetics	Study	Consumer	Normal
Female	24	Medical illustration	Expert	Producer	Normal
Male	23	computer science	Not interested	Consumer, Producer	Normal
Male	26	Biophysics	Expert	Producer	Normal
Male	22	informatics, protein engeneering	Study	Consumer, Producer	Normal
Male	29	Bioinformatics	Expert	Consumer	Normal
Male	36	Biomedical Visualization	High school	Producer	Normal

Table 1. Distribution of non-expert participants assigned the Detailed Static representation. The table shows gender, age, field of study/work, level of experience in biology and visualization, and color vision. Color gradient is used to indicate different fields of expertise (computer science-related, biology-related or irrelevant) and levels of experience (darker color indicates higher expertise). In the vision column, red color is used to highlight vision impairments. In this group, only one participant had a vision impairment.

**Abstract Static** (n=12)					
Gender	Age	Field of study/work	Biology Experience	Visualization Experience	Vision
Male	20	IT	Not interested	Consumer	Normal
Female	23	biochemistry	Expert	Consumer	Normal
Male	25	gamedev	High school	Consumer	Deuteranomaly
Male	27	Biomedical imaging	Expert	Producer	Normal
Female	20	Biology	Study	Consumer	Normal
Male	23	Computer science	High school	Producer	Normal
Male	22	Visual informatics	Study	Producer	Normal
Male	25	Visual Computing	Not interested	Producer	Normal
Female	22	Visual Informatics	High school	Consumer, Producer	Normal
Female	28	Visual informatics	High school	Consumer	Normal
Male	25	Law	High school	Consumer	Normal
Male	43	Law	High school	Not using	Normal

Table 2. Distribution of non-expert participants assigned the Abstract Static representation. The table shows gender, age, field of study/work, level of experience in biology and visualization, and color vision. Color gradient is used to indicate different fields of expertise (computer science-related, biology-related or irrelevant) and levels of experience (darker color indicates higher expertise). In the vision column, red color is used to highlight vision impairments. In this group, only one participant had visual impairment. The red frame indicates the participant whose quantitative scores were not used due to technical problems.

**Hybrid** (n=10)					
Gender	Age	Field of study/work	Biology Experience	Visualization Experience	Vision
Male	21	Informatics	High school	Consumer	Normal
Non-binary	23	Graphic design	Expert	Consumer, Producer	Normal
Male	21	Informatics- graphic design	High school	Consumer, Producer	Normal
Male	21	Computer science	High school	Consumer	Normal
Prefer not to say	23	Molecular biology	Expert	Consumer, Producer	Normal
Female	28	Visual Computing	Study	Consumer, Producer	Normal
Female	22	Computer vision - Digital image analysis and processing	High school	Producer	Normal
Female	23	Applied informatics- graphic design	High school	Consumer, Producer	Normal
Female	25	Human reproduction	Expert	Consumer	Normal
Female	22	Urban studies	High school	Consumer	Normal

Table 3. Distribution of non-expert participants assigned the Hybrid representation. The table shows gender, age, field of study/work, level of experience in biology and visualization, and color vision. Color gradient is used to indicate different fields of expertise (computer science-related, biology-related or irrelevant) and levels of experience (darker color indicates higher expertise). The red frame indicates the participant whose quantitative scores were not used due to technical problems.

**Narrated Video** (n=13)					
Gender	Age	Field of study/work	Biology Experience	Visualization Experience	Vision
Female	25	Andragogy	High school	Consumer	Normal
Male	28	Biochemistry	Expert	Consumer, Producer	Normal
Non-binary	20	Anthropology	Hobby	Consumer, Producer	Normal
Male	23	Game development	High school	Consumer	Normal
Male	32	Visualization	Study	Producer	Normal
Female	27	Veterinary medicine	Study	Consumer	Normal
Non-binary	28	Zoology	Study	Consumer	Normal
Male	26	Museum studies	High school	Not using	Normal
Male	28	Aircraft design	High school	Consumer	Normal
Female	22	Computer science	High school	Producer	Normal
Male	26	Forensics	Study	Consumer, Producer	Normal
Male	21	Computer science	High school	Consumer	Normal
Male	25	IT	Not interested	Consumer	Normal

Table 4. Distribution of non-expert participants assigned the Narrated Video representation. The table shows gender, age, field of study/work, level of experience in biology and visualization, and color vision. The color gradient is used to indicate different fields of expertise (computer science-related, biology-related or irrelevant) and levels of experience (darker color indicates higher expertise).

**Video** (n=11)					
Gender	Age	Field of study/work	Biology Experience	Visualization Experience	Vision
Female	24	Computer science	High school	Consumer	Normal
Female	21	Computer science	High school	Consumer, Producer	Normal
Male	36	computer science	High school	Producer	Normal
Male	22	Programming and development applications	High school	Not using	Normal
Male	25	Computer science	High school	Producer	Normal
Male	35	Molecular biology	Expert	Consumer, Producer	Normal
Female	24	Visualization (masters)	High school	Consumer, Producer	Normal
Male	23	Game development, computer science	High school	Consumer	Normal
Female	21	Computer science/ informatics	Study	Consumer	Normal
Male	23	Software engineering	Not interested	Consumer, Producer	Normal
Female	22	Computer science	High school	Consumer	Normal

Table 5. Distribution of non-expert participants assigned the Video representation. The table shows gender, age, the field of study/work, level of experience in biology and visualization, and color vision. The color gradient is used to indicate different fields of expertise (computer science-related, biology-related, or irrelevant) and levels of experience (darker color indicates higher expertise).
