# Supplementary material for: Seeing the unseen: Comparison study of representation approaches for biochemical processes in education
Source: PLoS One. 2023 Nov 6;18(11):e0293592. doi: 10.1371/journal.pone.0293592 (PMC10627439; doi:10.1371/journal.pone.0293592)
Supplement: S4 File — File containing the expert demographics and group division. (PDF) [file pone.0293592.s004.pdf]

S4 Supplementary Materials: Expert demographics and group division

**Experts** (n=9)						
Gender	Age	Field of research/ work	Years of experi-ence	Biology Experience	Visualization Experience	Vision
Female	39	Visualization	9	Collaborate	Expert	Normal
Female	39	Data visualization	12	Basic	Expert	Normal
Male	59	Proteomics	25	Expert	Collaborate	Normal
Male	43	Scientific visualization	11	Collaborate	Expert	Normal
Female	36	Chromatin organization	2	Expert	Basic	Normal
Male	60	Visualization	33	Collaborate	Expert	Normal
Male	35	Theoretical chemistry	12	Expert	Expert	Normal
Male	42	Visualization	18	Collaborate	Expert	Normal
Female	34	Biomedical visualization	10	Expert	Expert	Normal

Table 1. Distribution of the expert participants describing their gender, age, field of research/work, years of experience in the field, biology, and visualization level of experience, and color vision. The darker shades of colors indicate more experience in biology and visualization areas.
