# Supplementary material for: Seeing the unseen: Comparison study of representation approaches for biochemical processes in education
Source: PLoS One. 2023 Nov 6;18(11):e0293592. doi: 10.1371/journal.pone.0293592 (PMC10627439; doi:10.1371/journal.pone.0293592)
Supplement: S5 File — File containing the demographics description of the experts participating in the Focus Group. (PDF) [file pone.0293592.s005.pdf]

S5 Supplementary Materials: Demographic description of the experts participating in the Focus Group

**Focus Group** (n=5)		
Research/ work	Years of experience	Teaching experience
Biology/ chemistry high school teacher	4	Yes
Bio-visualization researcher	10	Yes
Visualization and human perception researcher	15	Yes
Graphic design, art and visualization expert	15	Yes
Biochemistry researcher	20	Yes

Table 1. An overview of the experts participating in the focus group and their experience. The first column describes the area of research/work, followed by years of experience in this field and teaching experience.
